# Automatic pseudo-coloring approaches to improve visual perception and contrast in polarimetric images of biological tissues

**DOI:** 10.1038/s41598-022-23330-6

**Published:** 2022-11-02

**Authors:** Carla Rodríguez, Albert Van Eeckhout, Enrique Garcia-Caurel, Angel Lizana, Juan Campos

**Affiliations:** 1 Optics Group, Physics Department, Universitat Autònoma de Barcelona, 08193 Bellaterra, Spain; 2 LPICM, CNRS, Ecole Polytechnique, Institut Polytechnique de Paris, 91120 Palaiseau, France

**Keywords:** Optics and photonics, Light responses, Imaging, Software, Image processing, Software

## Abstract

Imaging polarimetry methods have proved their suitability to enhance the image contrast between tissues and structures in organic samples, or even to reveal structures hidden in regular intensity images. These methods are nowadays used in a wide range of biological applications, as for the early diagnosis of different pathologies. To include the discriminatory potential of different polarimetric observables in a single image, a suitable strategy reported in literature consists in associating different observables to different color channels, giving rise to pseudo-colored images helping the visualization of different tissues in samples. However, previous reported polarimetric based pseudo-colored images of tissues are mostly based on simple linear combinations of polarimetric observables whose weights are set ad-hoc, and thus, far from optimal approaches. In this framework, we propose the implementation of two pseudo-colored methods. One is based on the Euclidean distances of actual values of pixels and an average value taken over a given region of interest in the considered image. The second method is based on the likelihood for each pixel to belong to a given class. Such classes being defined on the basis of a statistical model that describes the statistical distribution of values of the pixels in the considered image. The methods are experimentally validated on four different biological samples, two of animal origin and two of vegetal origin. Results provide the potential of the methods to be applied in biomedical and botanical applications.

## Introduction

Contrast in an image can be understood as the difference in either luminance (grey level) or color, that makes an object, or a region embedded in a portion of said image, to be distinguishable from the surrounding objects or regions. In biological samples, variations of luminosity or color of images are due to changes in the amount of light being absorbed, reflected or scattered by the probed sample. Since such fluctuations are related in a more or less involved different properties of the sample (composition, organization, order, structure….) then it can be said that absorption, reflection or scattering are sources of contrast that may allow to perceive variations in the physical properties of the imaged scene. The way in which a sample absorbs, reflects or scatters light not only depends on its intrinsic properties but also depends on the illumination conditions set to probe such sample. The simplest and maybe the most standard way of illuminating an object is to use partially coherent unpolarized, either polychromatic or monochromatic, light. Unfortunately in many cases, such a simple way of illumination may lead to poorly contrasted images which do not provide enough visual contrast. In those cases the use of more sophisticated methods of illumination, such as polarized light imaging, may be employed in order to boost the contrast in the images and therefore to retrieve information from probed samples with enhanced accuracy. The use of polarized light has been shown to be useful to reveal differences in the composition, thickness, density and structural organization as, for instance, in some tendinous structures in animal samples^1^ or some organelles in plants^2–4^. This is because, when illuminating these biological structures with polarized light, the light matter interactions lead to very different polarimetric properties (in retardance, dichroism and/or depolarization), which are directly related with their structural nature^1,5^. For instance, the anisotropic behavior of the plant vascular structures (mainly composed by organized and well-aligned cellulose chains^6–8^), or the animal structures built-in collagen (e.g., tendons), induce retardance to the polarization of the incident light beam.

One of the main advantages of polarization is the high sensitivity of the technique to differences in composition and structural organization within the inspected biological tissues. Among others, polarimetry allows the imaging of nerve fiber bundles in the human brain^9^ and the inspection of Alzheimer disease^10^. Polarization-related techniques also demonstrate high accuracy in the early detection of some cancers such as skin cancer^11^, colon cancer^12^, breast cancer^13,14^ and brain cancer^15^. Moreover, recent studies reveal the accurate performance of some machine learning algorithms built-in the polarimetric analysis of some biological tissues^16–20^. This situation may benefit the implementation of fast in-vivo and non-invasive pathology recognition methods, even at early stages. Analogously, the use of these optical techniques in plant science has proved to be very useful, as vegetal structures present polarimetric signatures (for instance, dichroism and birefringence) which can be potentially exploited for characterizing the spatial organization of some vegetal organelles such as the thylakoid membranes^2^ or the cell wall composition^8^. Recently, in addition to dichroism and birefringence, the use of depolarization observables to characterize structures in vegetal samples, is arousing growing interest^3,4^.

Keeping in mind the suitability of polarimetry for organic tissues characterization, it is feasible to achieve the overall enhancement of image contrast, including the revelation of regular intensity-hidden inherent structures, by conducting polarimetric measurements of biological tissues (either animal or vegetal). Polarimetric measurements result in a diversity of polarimetric figures (depolarization, diattenuation, polarizance, linear retardance, etc.)^21–23^ that may provide relevant information of tissue structures but which are commonly visualized as separated information channels. With the aim of enhancing the visualization of tissue imaging, recent works^1,24,25^ suggest the construction of a pseudo-colored image whose layers contain the different polarimetric features of the sample, i.e., different polarimetric information origins are included all together in a single enhanced image. The main idea in these works is to design a pseudo-colored function based on the weighted combination of three different polarimetric observables showing the highest image contrast between the structures of interest within the sample and associate each chosen observable to a primary color, RGB (red, green and blue). So far, the methods proposed to build polarimetric based pseudo-colored images are based on the qualitative (visual) selection of the polarimetric observables and their relative weights, and so, the final pseudo-colored model is quite arbitrary and not optimal in most of cases.

In this work we present, for the first time, two pseudo-coloring models which are designed based on two different methods that allow maximizing the visual contrast of different tissues in the sample. The two applied methods for building pseudo-colored images are based on: (1) the Euclidean distance between polarimetric values of different tissues; and (2) the Normal (Gaussian) function based on polarimetric data to estimate the probability of belonging to a particular tissue. Based on previous studies showing the special suitability of some depolarizing spaces in terms of biological samples discrimination^1,3–5^, we selected the Indices of Polarimetric Purity (IPPs)^26^ and the Components of Purity (CPs)^21^ spaces as the variables to be implemented within the pseudo-colored functions. Note that these two polarizing spaces are complementary, and fully describe the depolarizing properties of samples^27^. For completeness, we provide the comparison between pseudo-colored model results, based on the Euclidean and the Normal-based approaches, when analyzing different organic samples. Overall, images resulting from the pseudo-colored methods presented in this study overcome the regular polarimetric observables in terms of spatial location, visualization and recognition of the chosen structures within the inspected samples.

## Results

This section aims to show the results obtained with the Euclidean and Normal-based pseudo-coloring methods when used to inspect diverse types of biological samples (animal and vegetal samples). In both methods, the construction of the corresponding pseudo-coloring functions is based on the selection of a triplet of polarization-based figures or observables. As previously mentioned, we selected depolarization-related observables because as reported in literature^16,17,28–30^, and based in our previous experience we know that they are suitable to characterize biological samples^1,3–5^. To reinforce this argument, in section 1 of Supplemental document, we show the images of different biological structures related to a representative collection of different polarimetric observables used in the literature, as well as the images corresponding to IPPs and CPs, to show how the latter spaces give rise to greater visual contrast. In particular, two different scenarios were studied: (1) pseudo-coloring based on the Indices of Polarimetric Purity (IPPs, labeled as *P*_*1*_, *P*_*2*_ and *P*_*3*_); and (2) pseudo-coloring based on the Components of Purity (CPs, labeled as *P*, *D* and *P*_*S*_). These depolarization-based figures, which are discussed in detail in the “Methods” section, are selected here because they provide a complete description of the depolarization of light by the depolarization-related information of probed samples^27^. For illustrative purposes, in this section we show an application of the two image processing methods discussed here to four different types of samples, two of them were animal tissues and the other two were vegetal tissues. The two animal samples were biopsies from a lamb trachea (showing the trachea ring and the sheath), and a lamb tongue (showing the lingual papillae and epithelial tissue) respectively. The two vegetal tissues were taken from leaves of a specimen of *Quercus pubescens* presenting powdery mildew lesion caused by the fungus *Erysiphe alphitoides* (leaf powdery mildew vs leaf lamina), and, (4) leaves from a specimen of *Vitis vinifera* showing no symptoms of disease (leaf vein vs raphides vs cell cluster). Note that in the case of the *V. vinifera* leaf, we simultaneously analyzed three features (leaf vein/raphide/cell cluster) to study the ability of the proposed methods to characterize samples with more than two different features.

The pseudo-colored imaging construction procedure is summarized as follows. First, the experimental Mueller matrix (MM) image of all the studied samples was measured with a complete imaging polarimeter fully described in “Methods” section. From MM images, according to the mathematical background described in “Methods” section, we retrieved images of the depolarization-related observables corresponding to the polarimetric triplets of IPPs (*P*_*1*_, *P*_*2*_ and *P*_*3*_) and CPs (*P*, *D* and *P*_*S*_), i.e., six polarimetric images were derived from each experimental MM of a sample. From each one of these observables, we built four pseudo-colored images per sample, according to the approaches described in “Methods” section. These four pseudo-colored images, correspond to the Euclidean-based or Normal-based designs. In a final step the pseudo-colored images of each observable obtained with the two methods are compared to each other in order to select the one which higher contrast, and therefore the one which offers the cleared description of the studied sample. In the following, we provide the results obtained for the study of the above-stated biological samples. The analysis of the results is provided in the “Discussion” section.

### Polarimetric inspection of animal samples

**Figure 1:**
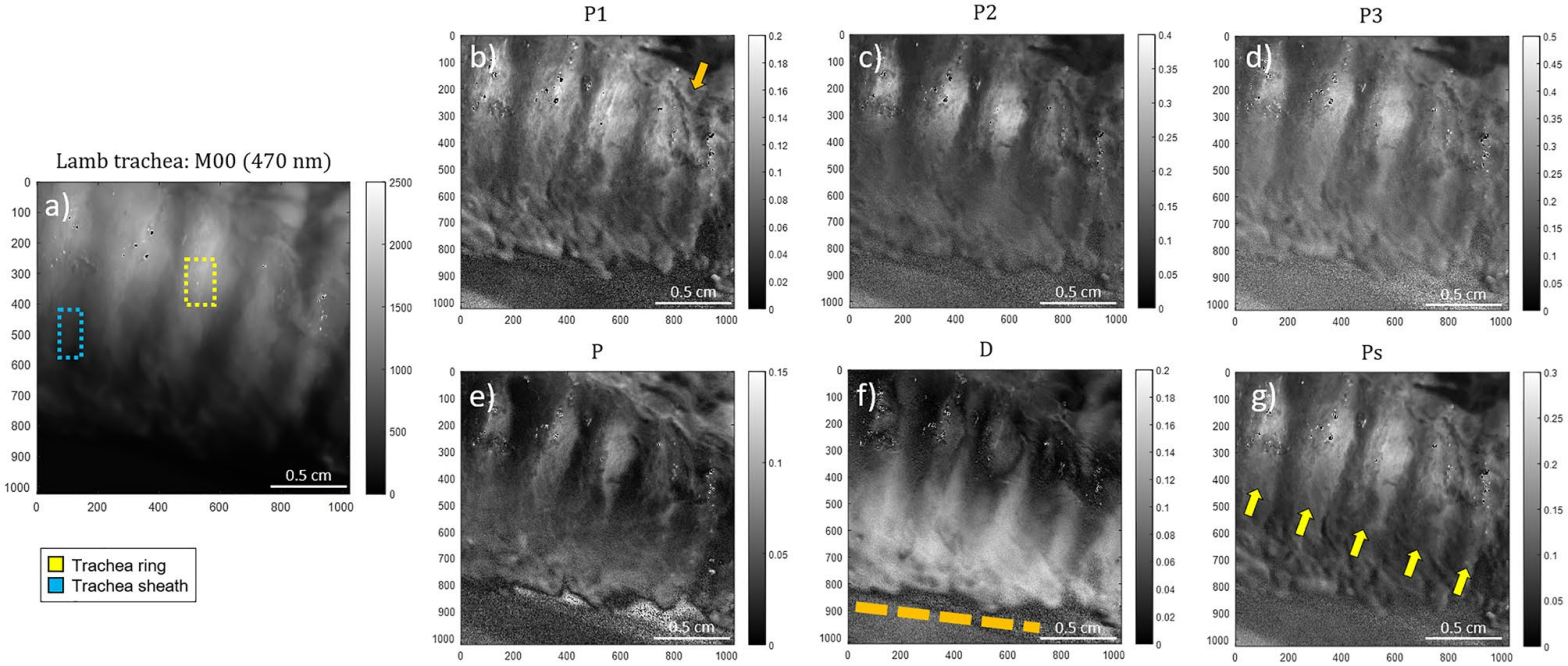
Images of a lamb trachea measured at 470 nm illumination wavelength: (**a**) unpolarized intensity image (*M*_*00*_), the Indices of Polarimetric Purity (**b**) *P*_*1*_, (**c**) *P*_*2*_ and (**d**) *P*_*3*_ and the Components of Purity (**e**) *P*, (**f**) *D* and (**g**) *P*_*S*_. The yellow dotted box (Fig. 1a) and yellow arrows (Fig. 1 g), indicate the location of the cartilaginous rings. The dotted blue box (Fig. 1a) indicates the location of the trachea sheath. The orange arrow (Fig. 1b) and the orange dotted line (Fig. 1f.) show the vascular structure within the external trachea sheath and the sample border, respectively.

The two animal samples discussed here correspond to a lamb trachea and a lamb tongue. The images shown are representative of the different images that we took from the same biopsy. The experimental data were taken in the scattering configuration, with a spectrally filtered blue light at 470 nm. The size of the probed samples was 2.2 × 2.2 cm^2^ and the images were taken using a camera with 1024 × 1024 pixels. Figure 1 shows the intensity image of the lamb trachea obtained with unpolarized light (*M*_*00*_, Fig. 1a; i.e., unpolarized intensity image) and the retrieved depolarization observables-based images corresponding to the IPPs (*P*_*1*_, *P*_*2*_, *P*_*3*_ in Fig. 1b–d) and CPs (*P*, *D*, *P*_*S*_ in Fig. 1e–g).

As previously mentioned, the following step is to construct pseudo-colored images based on the observables of the two chosen triplets of depolarization-based observables, the IPPs (*P*_*1*_*,* *P*_*2*_ and *P*_*3*_) and the CPs (*P*, *D* and *P*_*S*_), and to use them to apply the pseudo-color approach based on the Euclidean and the Gaussian approaches. Therefore, this procedure leads to four different pseudo-colored images for each sample studied (i.e., Euclidean model for (1) IPPs and (2) CPs plus Normal model for (3) IPPs and (4) CPs). In this subsection we focus on the results obtained for the animal samples (lamb trachea and lamb tongue samples).

**Figure 2:**
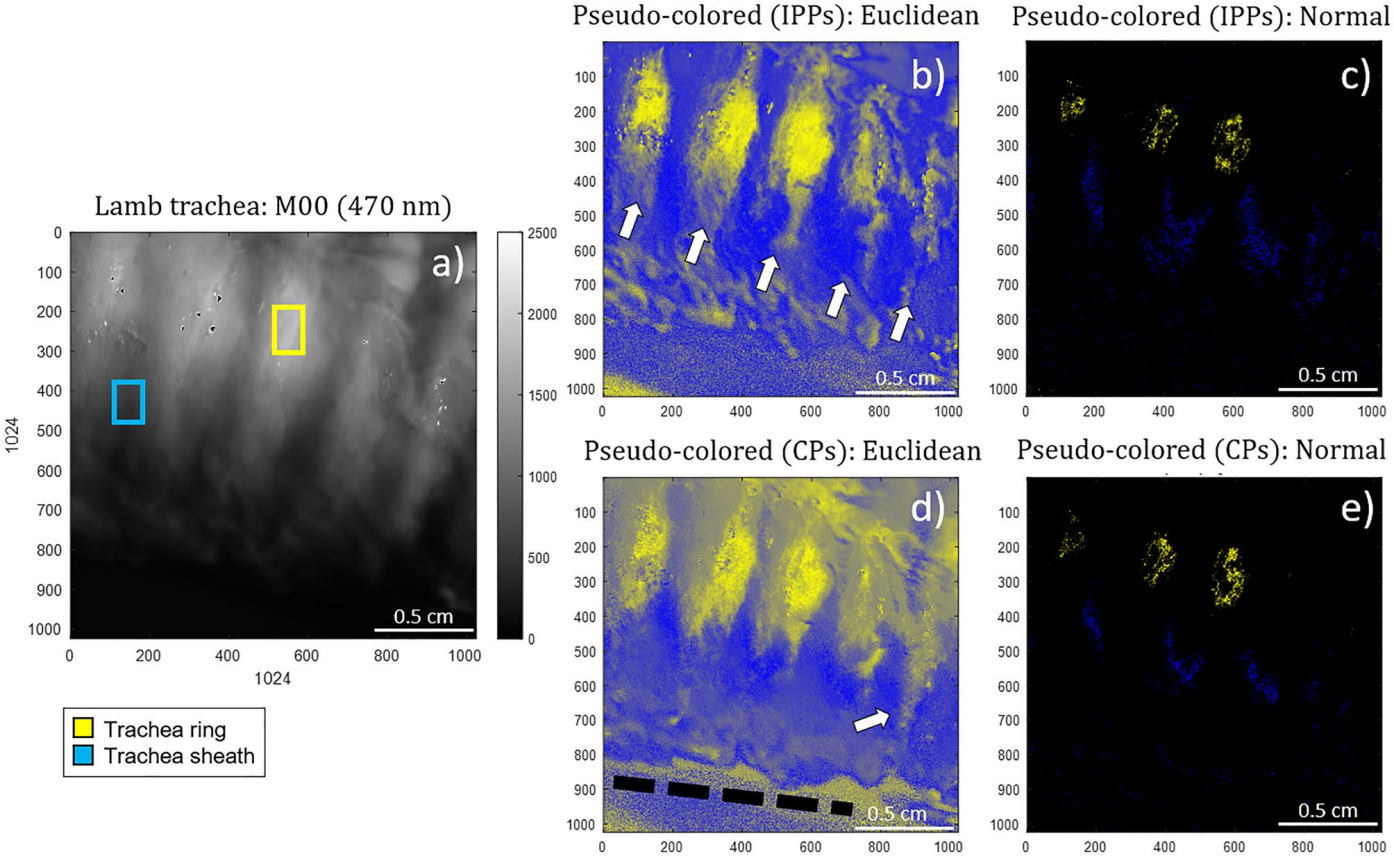
Raw and pseudo-colored images of the lamb trachea: (**a**) Unpolarized intensity image (*M*_*00*_) taken at 470 nm, (**b**) Euclidean and (**c**) Normal pseudo-colored images based on the IPP triplet, (**d**) Euclidean and (**e**) Normal pseudo-coloring based on the CP triplet. Yellow and blue squares show reference areas corresponding to the trachea ring and sheath, respectively. The white arrows (Fig. 2b and d) and the black dotted line (Fig. 2d) denote for the cartilaginous rings and the trachea border, respectively.

In the particular case of the lamb trachea sample, we choose the trachea cartilaginous rings (marked in yellow in Fig. 1a) and the sheath (marked in blue in Fig. 1a) as the features to be differentiated within the resulting pseudo-colored image. Under this scenario, we associate the trachea rings with the coordinates, $\overrightarrow {{C^{{{\text{Ring}}}} }} = [1,\;1,\;0]$ corresponding to the yellow color in the standard RGB color space. Analogously, the trachea sheath is associated with the RGB coordinates corresponding to the blue color, $\overrightarrow {{C^{{{\text{Sheath}}}} }} = [0,\;0,\;1]$. Figure 2 shows the pseudo-colored images of the lamb trachea in Fig. 1 resulting from the application of the two coloring approaches. The unpolarized image is also shown for visual comparison. The selected ROIs used for reference in the computations are indicated by the yellow and blue squares in Fig. 2a, respectively. Figure 2b and d correspond to the Euclidean pseudo-coloring for the polarimetric triplets IPPs and CPs, respectively. In counterpart, Fig. 2c and e illustrate the Normal pseudo-coloring for the polarimetric observables IPPs and CPs, respectively.

**Figure 3:**
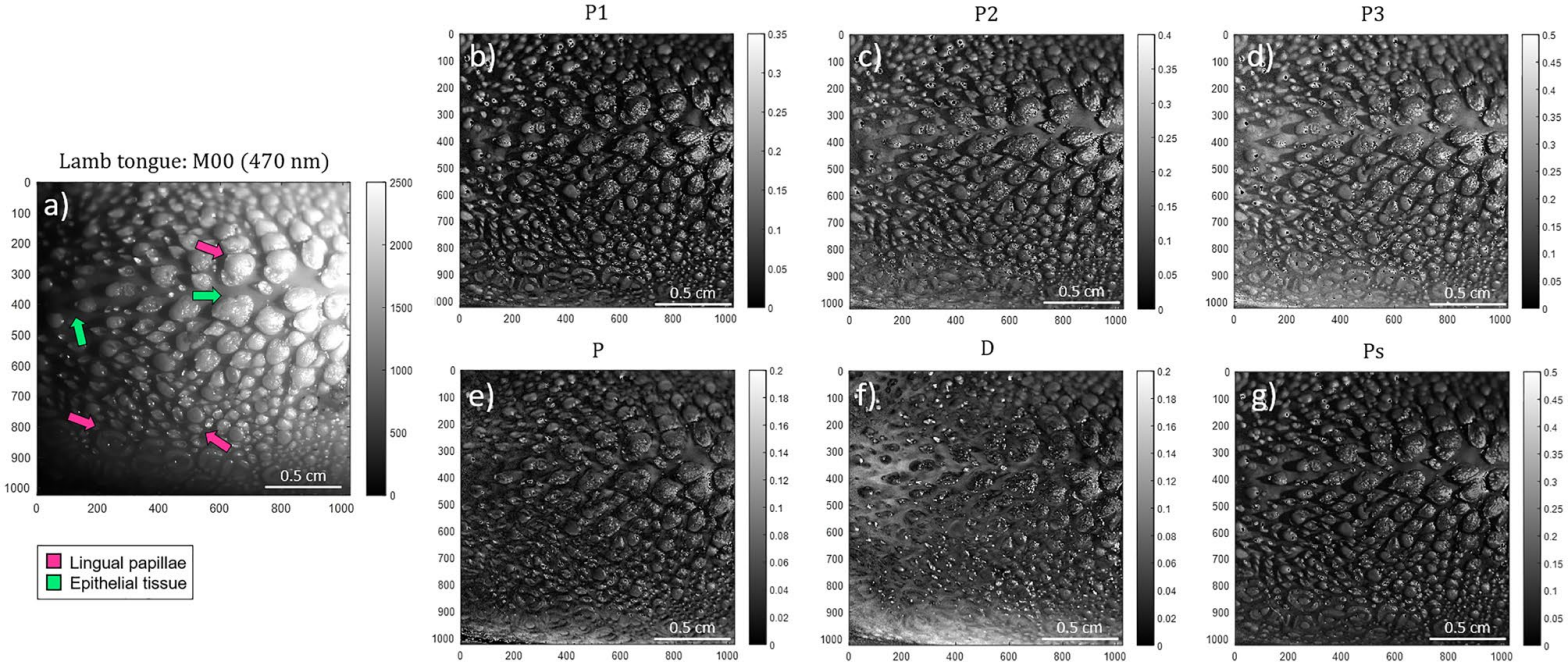
Polarimetric images of the lamb tongue measured at 470 nm illumination wavelength: (**a**) regular intensity image (*M*_*00*_), the Indices of Polarimetric Purity (**b**) *P*_*1*_, (**c**) *P*_*2*_ and (**d**) *P*_*3*_ and the Components of Purity (**e**) *P*, (**f**) *D* and (**g**) *P*_*S*_. The pink and lime-green arrows in Fig. 3a indicate the location of some of the lingual papillae and epithelial tissue regions, respectively.

Likewise, we apply the same polarimetric analysis above-described, but this time to the lamb tongue. Figure 3 presents typical images of the tongue (Fig 3a corresponds to the unpolarized diffuse reflectance, *M*_*00*_, and the corresponding polarimetric observables *P*_*1*_, *P*_*2*_, *P*_*3*_, *P*, *D* and *P*_*S*_ can be seen in Fig. 3b–g respectively).

**Figure 4:**
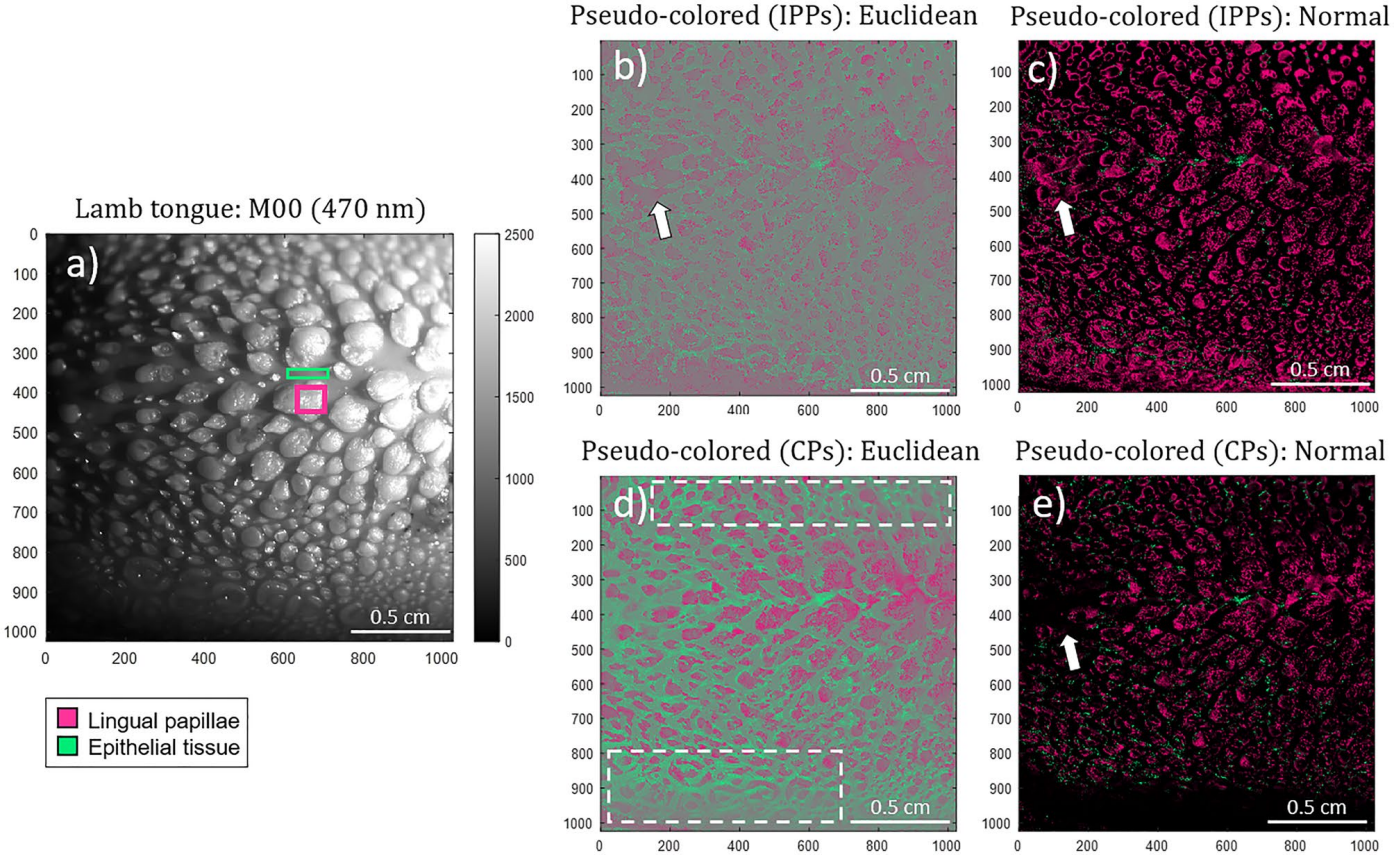
Intensity and pseudo-colored images for the inspected lamb tongue: (**a**) regular intensity image (*M*_*00*_) captured at 470 nm illumination wavelength, (**b**) Euclidean and (**c**) Normal pseudo-colored images for IPPs, (**d**) Euclidean and (**e**) Normal pseudo-coloring for CPs. Pink and green squares (Fig. 4a) denote for the selected regions of interest (ROI) of lingual papillae and epithelial tissue, respectively. White arrows (Fig. 4b, c and e) indicate a particular region only containing epithelial tissue. The dotted-squares indicate the unseen / out of focus region (by means of the unpolarized image, *M*_*00*_) of the tongue.

The two most well-differentiated characteristics within the lamb tongue correspond to the lingual papillae and epithelial tissue. Accordingly, we chose these features as the structures to be differentiated in the pseudo-colored images. In particular, we associate the polarimetric information of the lingual papillae with pink color with the following RGB coordinates $\overrightarrow {{C^{{{\text{Papillae}}}} }} = [1,\;0,\;0.5]$, and the epithelial tissue with the RGB coordinates corresponding to lime-green color with the RGB coordinates $\overrightarrow {{C^{{{\text{Epithelial}}}} }} = [0,\;1,\;0.5]$, (both arrowed with their corresponding color in Fig. 3a). Figure 4 shows the diffuse reflectance image *M*_*00*_ (Fig. 4a) and the images resulting from the implemented pseudo-colored functions. The ROIs corresponding to lingual papillae and epithelial tissue are indicated in Fig. 4a with pink and lime-green squares, respectively. Figure 4b and d correspond to the Euclidean pseudo-coloring of the inspected sample for the depolarization spaces IPPs and CPs, respectively. Besides, the Normal pseudo-coloring for the polarimetric triplets IPPs and CPs is presented in Fig. 4c and e, respectively.

### Polarimetric inspection of vegetal samples

**Figure 5:**
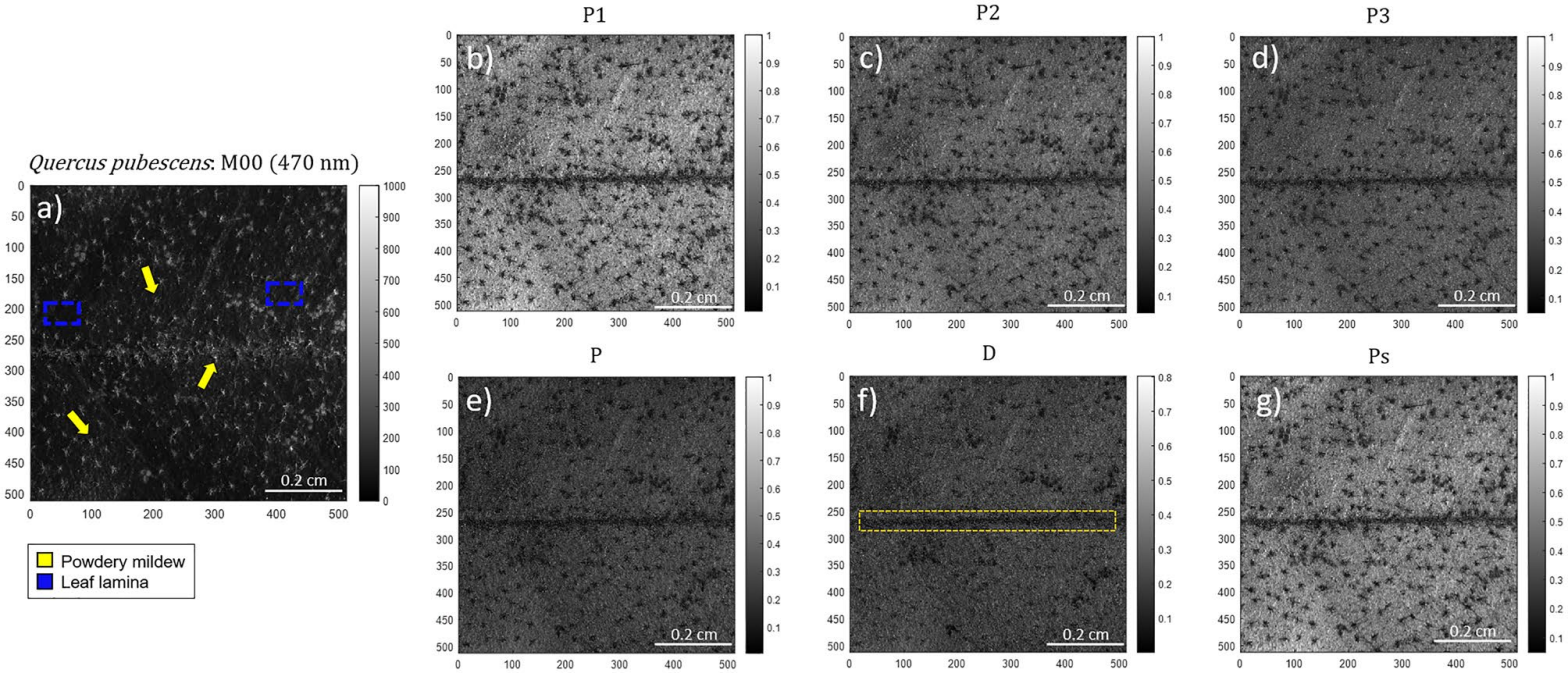
Polarimetric images of the Quercus pubescens leaf measured at 470 nm illumination wavelength: (**a**) regular intensity image (M_00_), the Indices of Polarimetric Purity (**b**) P_1_, (**c**) P_2_ and (**d**) P_3_ and the Components of Purity (**e**) P, (**f**) D and (**g**) P_S_. The yellow arrows, and the blue dotted box (both in Fig. 5a) denote for some of the powdery mildew lesions and the leaf lamina location, respectively. The orange-dotted box (Fig. 5f.) indicates the location of the leaf vein.

In this section we discuss the results obtained from two different plants; one was a leaf of *Q. pubescens* and the other was a leaf of *V. vinifera*. The leaf of *Q. pubescens* show powdery mildew lesions caused by the fungus *E.rysiphe alphitoides* while the leaf of *V. vinifera* did not show any sign of infection or parasitic invasion. The leaf *Q. pubescens* was measured with the same imaging polarimeter used to obtain the images discussed in the previous section. The typical size of the field of view of the images measured with such polarimeter is1.1 × 1.1 cm^2^ (512 × 512 pixels). The experimental Mueller matrices of the leaf of *V. vinifera* were measured with a polarimetric microscope in transmission at a wavelength of 533 nm. The field of view of the images taken by the microscope correspond to a circle of radius 100 µm. The non-polarized (transmission, diffuse reflectance) intensity image (*M*_*00*_) and the retrieved depolarization observables (*P*_*1*_, *P*_*2*_, *P*_*3*_, *P*, *D* and *P*_*S*_) for the *Q. pubescens* sample are presented in Fig. 5.

**Figure 6:**
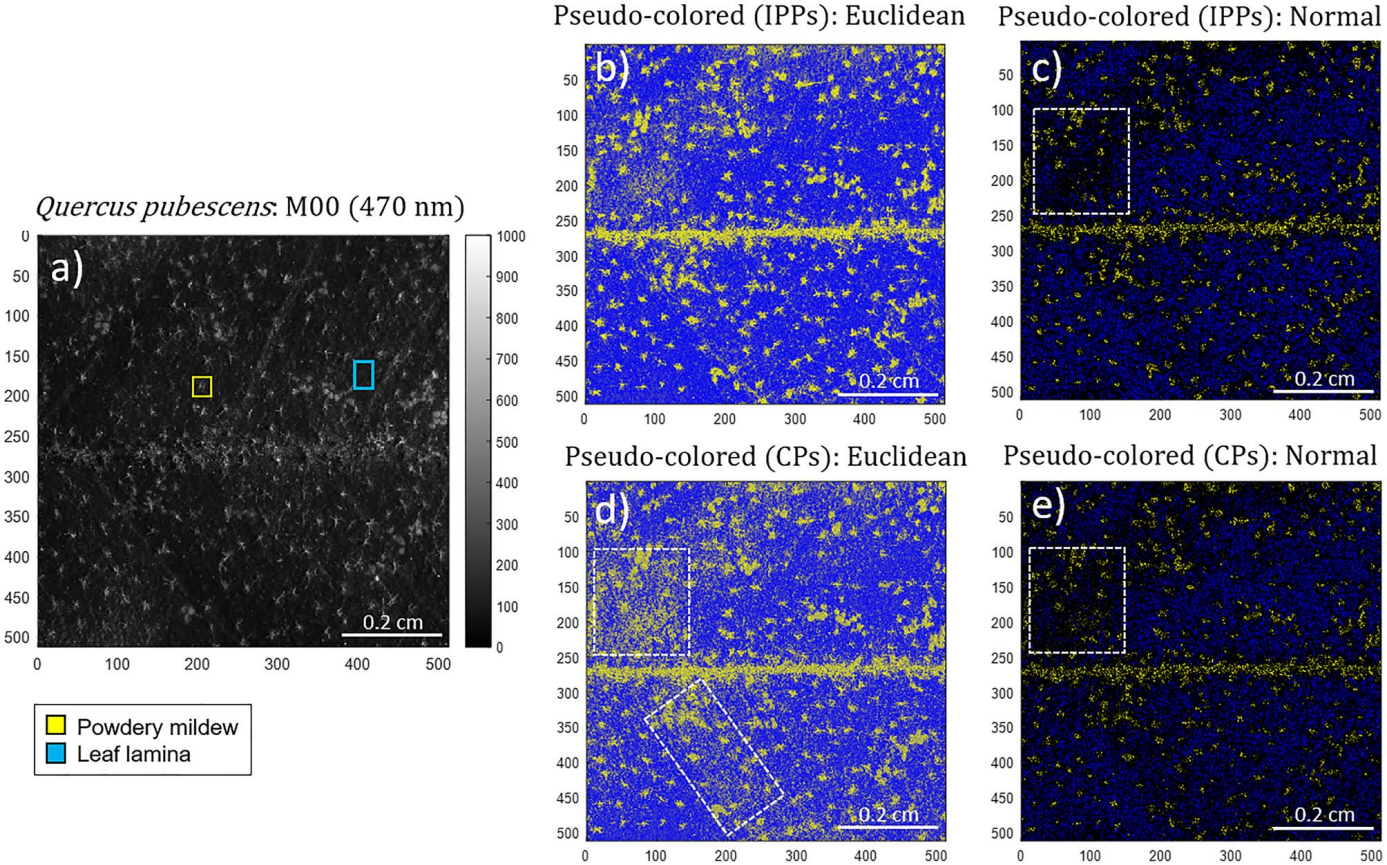
Intensity and pseudo-colored images for the inspected *Quercus pubescens* leaf: (**a**) non-polarized (transmission—diffuse reflection) image (*M*_*00*_) captured at 470 nm illumination wavelength, (**b**) Euclidean and (**c**) Normal pseudo-colored images for IPPs, (**d**) Euclidean and (**e**) Normal pseudo-coloring for CPs. Yellow and blue squares (Fig. 6a) denote for the selected regions of interest (ROI) of powdery mildew lesion caused by the fungus *Erysiphe alphitoides* and the healthy leaf lamina, respectively. White-dotted squares (Fig. 6c and e) denote for the misrecognized pixel regions of both Euclidean and Normal-based methods.

In order to explore the ability of pseudo-colored images to enhance the visual contrast between healthy and infected areas of the leaf, we chose to inspect the characteristics corresponding to the powdery mildew lesions and the healthy leaf lamina. In particular, we associate the polarimetric information of the powdery mildew with the yellow color, with RGB coordinates $\overrightarrow {{C^{{{\text{Powdery}}}} }} = [1,\;1,\;0]$, and the leaf lamina with blue color with RGB coordinates, $\overrightarrow {{C^{{{\text{Lamina}}}} }} = [0,\;0,\;1]$. Note that the chosen colors, blue and yellow are complementary to each other, therefore they even the slightest shadow of them can be can be visually discriminated with ease. Figure 6 presents the visual comparison between the *M*_*00*_ image (Fig. 6a) and the four pseudo-colored images based on polarimetric observables for the inspected leaf of *Q. pubescens*. As discussed in previous section the pseudo-colored images were obtained using the functions described in Methods with the above-stated $\overrightarrow {{C^{{{\text{Powdery}}}} }}$ and $\overrightarrow {{C^{{{\text{Lamina}}}} }}$ values. The selected regions of interest (ROI) corresponding to powdery mildew and leaf lamina, are indicated in Fig. 6a with yellow and blue squares, respectively. The resulting Euclidean and Normal pseudo-coloring for the polarimetric spaces IPPs and CPs are shown in Fig. 6b and d and Fig. 6c and e, respectively.

**Figure 7:**
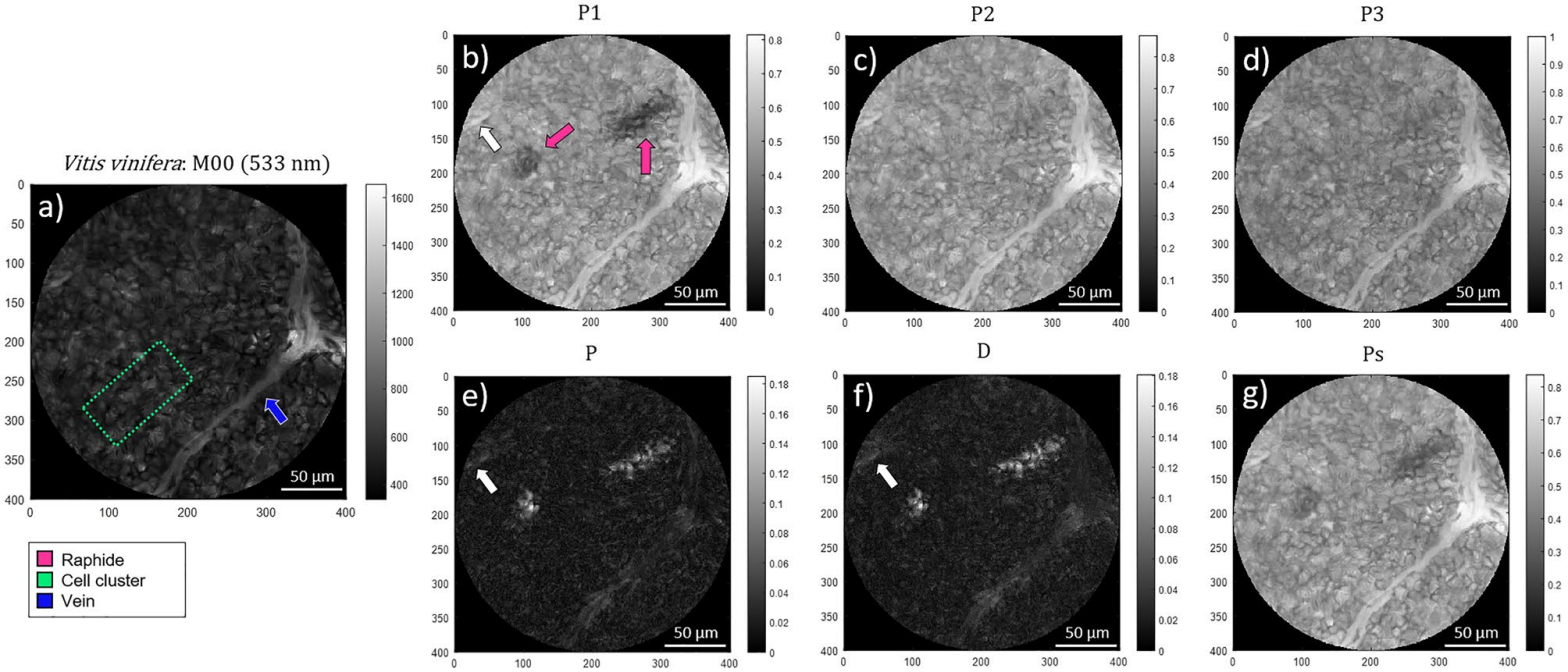
Polarimetric images of the *Vitis vinifera* leaf measured under the microscope for 533 nm illumination wavelength: (**a**) non-polarized transmission image (*M*_*00*_), the Indices of Polarimetric Purity (**b**) *P*_*1*_, (**c**) *P*_*2*_ and (**d**) *P*_*3*_ and the Components of Purity (**e**) *P*, (**f**) *D* and (**g**) *P*_*S*_. The blue and pink arrows (Fig. 7a, and b) indicate the location of the vein and the raphides, respectively. The white arrows (Fig. 7b, e and f) indicate the secondary vascular structure. The lime-green dotted box indicates an illustrative region comprising a cell cluster.

Regarding to the inspection of *V. vinifera* leaf, we chose to differentiate three main structures present in the leaf, the raphides, the vegetal cells walls of the leaf lamina and the venous system, each one associated with the pink, lime-green and blue colors corresponding to the RGB coordinates $\overrightarrow {{C^{{{\text{Raphide}}}} }} = [1,\;0,\;0.5]$, $\overrightarrow {{C^{{{\text{Cell}}}} }} = [0,\;1,\;0.5]$, and $\overrightarrow {{C^{{{\text{Vein}}}} }} = [0,\;0,\;1]$, respectively. Figure 7 presents the regular intensity image (*M*_*00*_) and the retrieved depolarization observables (*P*_*1*_, *P*_*2*_, *P*_*3*_, *P*, *D* and *P*_*S*_) for the *V. vinifera* leaf.

**Figure 8:**
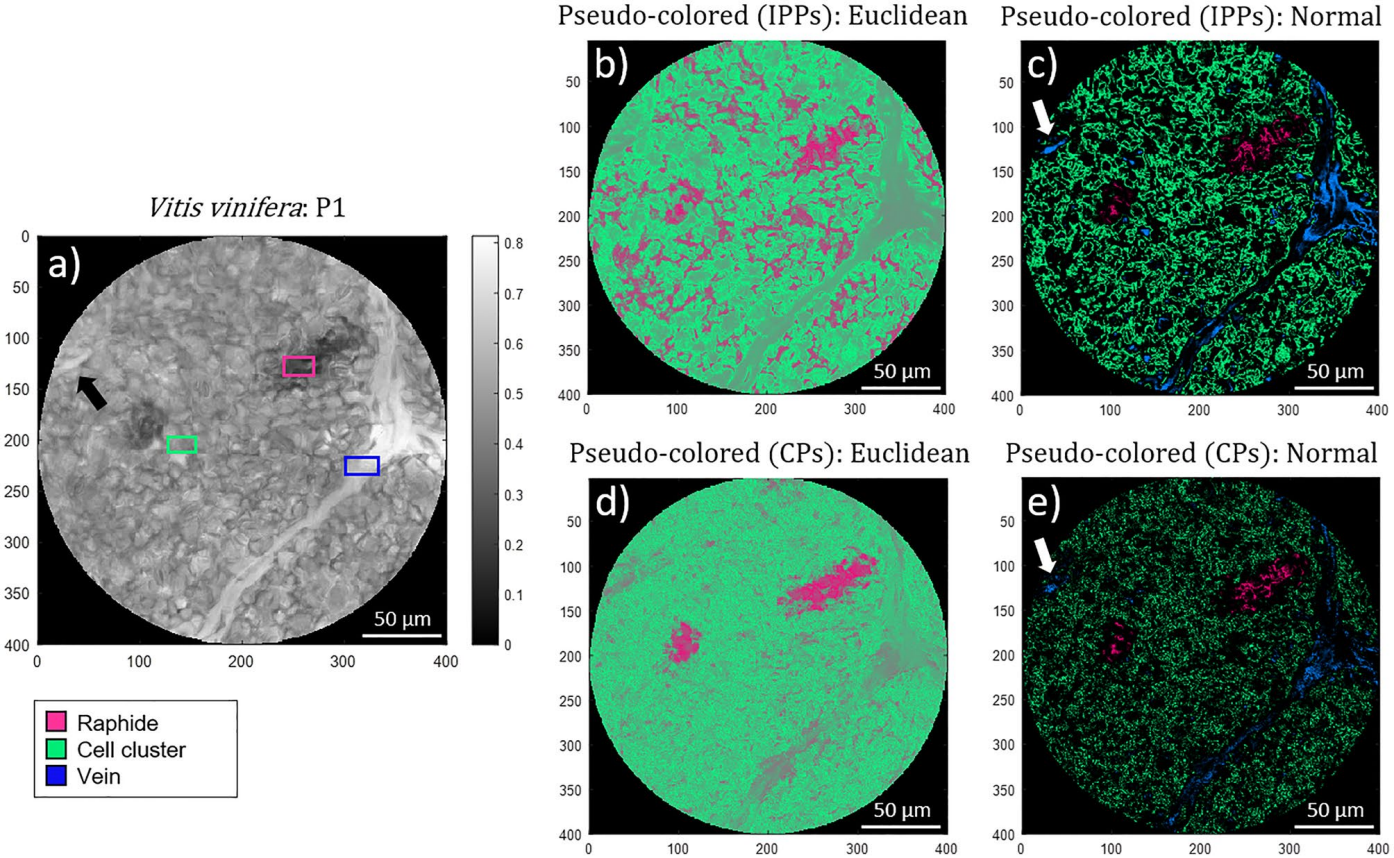
Non-polarized transmission (*M*_*00*_) and pseudo-colored microscopic images from a *Vitis vinifera* leaf section captured at 533 nm illumination wavelength: (**a**) polarimetric purity index *P*_*1*_, (**b**) Euclidean and (**c**) Normal pseudo-colored images for IPPs, (**d**) Euclidean and (**e**) Normal pseudo-coloring for CPs. Pink, lime-green and blue squares denote for the selected regions of interest (ROI) of raphides, leaf lamina cells and vein, respectively. The white arrows indicate the location of the secondary vascular structure.

Since the Euclidean distances approach is valid for just two classes, we decide to choose cell walls of the leaf lamina. In contrast, the Normal approach accepts more than two classes, so with this method we can visualize, simultaneously, the three above-mentioned features: the raphides, the leaf lamina cells and the vein. In this case, the pseudo-colored approach results in the images shown in Fig. 8. This example allows us to show the ability to recognize tissues that are invisible in non-polarimetric images thanks to the use of polarimetric images. This is because some biological structures analyzed are invisible in the intensity image (Fig. 7a) and therefore, the ROIs used to train the model are directly obtained from polarimetric channels, and in particular, from the *P*_*1*_ image which was the one giving the largest visual contrast. In particular, Fig. 8 presents a visual comparison between the image corresponding to the polarimetric purity index *P*_*1*_ (Fig. 8a) and the resulting images from both the Euclidean and the Normal-based pseudo-coloring methods for the *V. vinifera* inspected section. As said, note that unlike previous cases, in this case we select the polarimetric purity index *P*_*1*_ (Fig. 8a) as reference instead of the intensity image (Fig. 7a). This is because some structures of interest (the raphides in this case) are visible thanks to the *P*_*1*_ channel, but completely invisible in the intensity imae (Fig. 7a), and therefore, corresponding ROIs were obtained from Fig. 8a. For the sake of clarity, the ROIs of the structures corresponding to the raphide, cell cluster and leaf vein, are indicated in Fig. 8a with pink, lime-green and blue squares, respectively. Figure 8b and d illustrate the Euclidean pseudo-coloring of the sample by means of the IPPs and CPs observables, respectively. Figure 8c and e show the Normal pseudo-coloring for the IPPs and CPs implementation, respectively.

## Discussion

In the following, we present a discussion regarding the pseudo-colored images of different animal tissues presented in the previous “Results” section. In this work, we choose the polarimetric channels that give larger contrast between tissues of interest, and apply them within diverse pseudo-coloration methods. Note that we do not select the intensity image as one channel for the pseudo-coloration approach. The loss of the intensity channel is a choice in our case, in favor of a more adequate space to enhance the contrast between different tissues. We use the RGB color space mainly because it is made up of three orthogonal variables that vary from 0 to 1 and the IPPs are restricted to the same value range. However, we want to note that the IPPs are not completely independent since the value of *P*_*3*_ channel determines the range of *P*_*2*_ and *P*_*1*_^21^. For this reason, another color composition may also be useful (or more optimal) to represent the IPPs space.

In the case of the lamb trachea sample (Fig. 1), the two most well-differentiated structures for the lamb trachea in the non-polarized reflectance image (Fig. 1a) correspond to the cartilaginous rings, composed by hyaline cartilage, and the external sheath, also known as tunica adventitia, mainly composed by collagen (both structures are indicated in Fig. 1a with yellow and blue squares, respectively). These main differences in tissue composition give raise to the different polarimetric responses explored and discussed in this work. Figure 1 clearly shows how the images corresponding to the enpolarization metrics (*P*_*1*_, *P*_*2*_, *P*_*3*_, *P*, *D* and *P*_*S*_ in Fig. 1b–e) point well the difference between the two types of tissues and also present an enhanced contrast when compared to the non-polarized diffuse reflectance (*M*_*00*_ in Fig. 1a). Regardless of the model used to assign colors to pixels, the key to ensure a vivid contrast enhancement using a pseudo-colored approach from several channels is the fact that a given channel must be sensitive to characteristics of the sample that the other channels are not. For instance, in the particular case of the trachea, *P*_*1*_ provides information about the surface details of the trachea sheath (e.g. some vascular structures within the external sheath, indicated with the orange arrow in Fig. 1b), while *P*_*2*_ and *P*_*3*_ are sensitive to the cartilaginous rings (Fig. 1c–d). Interestingly, the cartilaginous rings demonstrate higher mean values for IPPs (*P*_*1*_ = 0.14 ± 0.01, *P*_*2*_ = 0.27 ± 0.01 and *P*_*3*_ = 0.37 ± 0.01) when compared with the trachea sheath (*P*_*1*_ = 0.05 ± 0.01, *P*_*2*_ = 0.10 ± 0.01 and *P*_*3*_ = 0.18 ± 0.01), this meaning that the cartilaginous rings are less depolarizing than the trachea sheath. Accordingly, cartilaginous rings demonstrate higher mean polarizance *P* (Fig. 1e) values (*P* = 0.06 ± 0.01) than the sheath (*P* = 0.02 ± 0.01). In contrast, the sheath induces more diattenuation (*D* = 0.10 ± 0.01) than the rings (*D* = 0.05 ± 0.01) (Fig. 1f). Note that in all the cases, polarizance and diattenuation channels present values lower than 0.1. This situation indicates that the trachea can be considered as a non-dichroic structure and that the observed depolarizing effects can be mostly associated with either multiple scattering or fluctuations in the value and the direction of the retardance of birefringent structures present in cartilaginous rings and the trachea sheath. In this regard, retardance is encoded in the sphericity degree *P*_*S*_ and, as shown in Fig. 1g, it highlights the details of the trachea rings (mean value of *P*_*S*_ = 0.2 ± 0.01, see the yellow arrows in Fig. 1g) in addition to the surface of the sheath (mean value of *P*_*S*_ = 0.07 ± 0.01). Interestingly, note that the IPPs provide information about the structure of the polarimetric randomness of samples, and that they are directly connected with the CPs through the relation^27^: $P^{2} + D^{2} + 3P_{s}^{2} = 2P_{1}^{2} + \frac{2}{3}P_{2}^{2} + \frac{1}{3}P_{3}^{2}$. This fact, together with the above-discussed low values for *P* and *D* in the trachea sample, suggests that high values of *P*_*S*_ may be related to the fact that the cartilaginous structure induces less depolarization according to the strong alignment of collagenous fibers. In contrast, the low *Ps* value of the sheath demonstrates the higher depolarizing behavior of that tissue. Importantly, all the polarimetric observables unveil the trachea borders (see the orange dotted line in Fig. 1f) not seen in the intensity image (Fig. 1a).

Regarding to the pseudo-colored images, the Euclidean distance-based method for both polarimetric triplets of IPPs and CPs (Fig. 2b and , respectively) enhances the perceived contrast between cartilaginous rings and trachea sheath. Conversely, the Normal-based approach (based on IPPs and CPs in Fig. 2c and e) has not enough capability to improve visualization, as most pixels in the image are not recognized as part of any of the two classes (i.e., the probability, calculated through the normal function, of belonging to either the ring or the sheath is almost zero). Therefore, according to Eq. (10) in “Methods” section, those pixels are represented in black. Concerning the observables used to implement the pseudo-coloring model based on Euclidean distances, the use of the IPPs set of observables seem to well differentiate the five rings (indicated with white arrows in Fig. 2b) from the sheath, thus allowing a clear an accurate identification and spatial localization of the cartilaginous tissue within the sample. In turn, the same pseudo-coloring model based on the CPs set of observables (Fig. 2d) also succeeds to give a clear discrimination between the cartilaginous rings and the trachea sheath but the transition between these two classes is less accurate than in the IPPs case (i.e., the trachea sheath occupying the space between rings is misrecognized as cartilaginous tissue too). Despite this, the fifth trachea ring (white arrow in Fig. 2d) and the full trachea structure border (black dotted line in Fig. 2d) are both better spatially located in the CPs case. In summary because the poor ability of the coloring based on the Normal model to discriminate among different classes, the most adequate method to visually inspect structures within the lamb trachea sample is the Euclidean distances approach.

The second case of study is the lamb tongue. Likewise to the case of the lamb trachea above-discussed, we retrieve, from the experimental Mueller matrix measurement of the lamb tongue, the isolated polarimetric observables images (*P*_*1*_, *P*_*2*_, *P*_*3*_, *P*, *D* and *P*_*S*_). In this sample, we focus on two tissue classes: the lingual papillae and the epithelial tissue (some of those are indicated with pink and green-lime arrows in Fig. 3a, respectively). Regarding the depolarizing content, the mean values for lingual papillae and epithelial tissue are: *P*_*1*_=0.09±0.01, *P*_*2*_=0.14±0.01, *P*_*3*_=0.21±0.01, and, *P*_*1*_=0.03±0.01 (Fig. 3b), and *P*_*2*_=0.10±0.01 (Fig. 3c) and *P*_*3*_=0.20±0.01 (Fig. 3b,c and d), respectively. In overall, both the lingual papillae and the epithelial tissue demonstrate similar low IPPs values, which translate into the tissues having high depolarizing capability. In addition, the epithelial tissue and lingual papillae also show similar (and low) mean polarizance *P* values: *P*=0.01±0.01 and *P*=0.04±0.01, respectively (Fig. 3e). In turn, the diattenuation *D* becomes higher at sample borders (see the bottom region in Fig. 3f), reaching mean values of *D*=0.13±0.01 compared with the *D*=0.05±0.01 of the central region. The values of polarizance and diattenuation obtained for the lingual papillae and epithelial tissue demonstrate that they are mostly non-dichroic structures. Therefore, depolarization observed can be associated to either the effect of multiply scattered light or the fluctuation in the value and the direction of the birefringent structures. As in the case of the trachea sample, due to this non-dichroic behavior, the *P*_*S*_ values obtained for the tongue demonstrates more depolarizing behavior for the epithelial tissue (lower values of *P*_*S*_=0.08±0.01) than for the papillae (*P*_*S*_=0.12±0.01), this leading to a visual contrast between these two classes (Fig. 3g). Importantly, we want to highlight a significant improvement provided by some of the depolarizing channels (Fig. 3b–g) with respect to non-polarized diffuse reflectance image (Fig. 3a). In particular, note that the tongue structures placed at the bottom part of the non-polarized diffuse reflectance image are very difficult, or even impossible to be seen in some parts (check for instance the region between the Y axis pixels 800 and 1000) due to the low contrast between them. Same effect can be observed at the very top of the image. This is mostly due to intensity losses or defocusing introduced by the measure of a non-planar sample (the tongue), where only the central region is properly illuminated and in focus. In contrast to that, a clear visual enhancement of those regions is provided by depolarization observables, specially by *P*_*1*_ and *P*_*S*_ channels, in which the lingual papillae and the epithelial tissue are clearly observed in the whole image. The latter may be explained by the fact that contrast in polarimetric images is less sensitive to focus than in non-polarized intensity images^3^ and thus, lead to this image improvement. Indeed, while a fine tune of focus increases sharpness, and thus contrast in non-polarized intensity images, the relative difference between the polarimetric response of adjacent zones is what provides contrast in a polarization-based image^3^. This situation is one of the reasons that explains the interest of using polarimetric methods for tissue characterization.

Afterwards, we implement the pseudo-coloring functions for the lamb tongue sample. Accordingly, the lingual papillae and the epithelial tissue were associated with two different colors: pink and lime-green, respectively. We see how the best results, in terms of visualization, is obtained for the Euclidean pseudo-coloring method based on the CPs triplet (Fig. 4d). In agreement with the discussion related to previous Fig. 3, note how tongue structures out of focus are now well identified and discriminated in the pseudo-colored images, as they are based on polarimetric observables (see Fig. 3b–g) not in intensity gray levels such as the non-polarized diffused reflectance observable (Fig. 3a). In the case of the pseudo-coloring based on the Normal method (Fig. 4c and e), the best visualization is obtained for the implementation based on the IPPs triplet (Fig. 4c). However, the class recognition rate is lower than for the Euclidean distance case (see for instance white arrows in Fig. 4c and e, pointing the lingual papillae recognition for the Normal method, compared with correct recognition of the epithelial tissue in Fig. 4b, by the Euclidean distance method). In the case of the pseudo-coloring based on the Normal method, the mean values selected for the epithelial tissue (obtained from the selected ROIs, green rectangle in Fig. 4a) are not representative enough of the properties of the epithelial tissue, which present a large variance across the image. For this reason, an important part the epithelial could not be successfully assigned to the correct class by the method and therefore appear in black in the pseudo-colored images (Fig. 4c and e). Under this scenario, it is shown the pseudo-coloring based on the Euclidean approach performs better than the pseudo-coloration based in the Normal approach. In order to improve the performance of the pseudo-coloring based in Normal methods, it may be possible, for instance, to select multiple ROIs of each class tissue across the image and then to evaluate the corresponding mean values. Unfortunately, the latter may result in an obvious complication of the method which is to be compared to the simplicity shown by the Euclidean approach (one single ROI assignment per tissue class).

In the following, we switch to the inspection of plant tissues. Following the same protocol that we applied to animal tissues in previous cases, we measured the experimental Mueller matrix corresponding to a leaf of a specimen of *Q. pubescens* showing powdery mildew lesions due to the infection of the fungus *E. alphitoides*. Accordingly, the two classes to be differentiated within the sample correspond to the healthy tissue (leaf lamina) and the regions showing symptomatology (powdery mildew). For the sake of clarity, in Fig. 5a we present the intensity image of the leaf, and we highlight some of the locations corresponding to the lesions caused by the pathogen (yellow arrows) and the regions corresponding to healthy tissue (blue dashed squares). The evident differences in biological structure and chemical composition between a plant tissue, the leaf of *Q. pubescens*, and a fungus, the *E. alphitoides,* lead to different polarimetric responses. The polarimetric images based on the IPPs (Fig. 5b–d) demonstrate the enhancement of the overall image contrast, thus allowing a proper spatial localization of the leaf lesions. Among the IPPs, *P*_*1*_ is the observable that leads to larger contrast between classes, and demonstrates a higher capability to discriminate between features (Fig. 5b), followed by *P*_*2*_ and *P*_*3*_ (Fig. 5c and d, respectively). Regarding the depolarization content of the inspected sample, the healthy leaf lamina shows high IPP mean values: *P*_*1*_=0.82±0.01, *P*_*2*_=0.89±0.01 and *P*_*3*_=0.99±0.01. Conversely, the powdery mildew shows lower mean values: *P*_*1*_=0.27±0.01, *P*_*2*_=0.43±0.01 and *P*_*3*_=0.60±0.01. Accordingly, it can be said that the leaf lamina induces less depolarization to the incident light than the powdery mildew lesions. The low depolarizing performance of the leaf lamina suggests that it presents a well-organized cell layout within the leaf, and a homogeneous polarization response through the structure. In contrast, the effect of the fungus seems to modify the cell layout structure of the leave, leading to an evident modification of the polarimetric response of the regions with lesions. This different polarimetric response between healthy and infected regions may also be observed in the polarizance *P* channel (Fig. 5e): the healthy lamina shows mean polarizance value of *P*=0.35±0.01 and the powdery mildew of *P*=0.12±0.01. Note that values of polarizance larger than 0.3 show a non-negligible dichroic response of the vegetal cells in the *Q. pubescens* leaf, being this dichroic capability significantly reduced after sample infection. Regarding to the diattenuation response (Fig. 5f), the sample demonstrates, overall, low mean values (*D*=0.13±0.01 and *D*=0.23±0.01 for leaf lamina and powdery mildew, respectively). Finally, among the presented CPs, the sphericity degree *P*_*S*_ is the observable whose performance better enhances the image contrast between the inspected features (see Fig. 5g): healthy lamina shows mean value of *P*_*S*_=0.86±0.01 and powdery mildew, *P*_*S*_=0.34±0.01. The differences in *P*_*S*_ values of the healthy and infected regions may be due to modifications in the cell layout organization (cell alignment) and/or within their polarimetric behavior (mainly birefringence or polarizance features). Furthermore, it is important to mention that the black horizontal line (indicated with the orange botted box in Fig. 5f) corresponds to an underlying principal vein of the leaf.

Concerning the pseudo-coloring of the image of the *Q. pubescens* leaf, we associate the yellow and blue colors to the powdery mildew and leaf lamina classes respectively. When using the pseudo-coloring method based on the Euclidean distance approach (Fig. 6b and d) an obvious visual enhancement of the studied classes is obtained. The infected regions by the powdery mildew clearly appear in a shiny yellow over a blue background which corresponds to the of the leaf lamina. The use of the IPPs set of observables seem to provide a better discrimination than the CPs set. The latter can be seen, because in the pseudo-colored image using the CPs set (Fig. 6d) there are some areas (highlighted with white rectangles) where some pixels are misclassified while the same pixels are correctly classified in the image pseudo-colored using the IPPs set. Analogously to the conclusions found for biological samples previously discussed, the pseudo-coloring method based on the Euclidean distance method shows a better performance than the method based on the Normal function-based approach. More in detail, for the particular case of the *Q. pubescens*, the set of observables that gives the most robust results in terms of discrimination efficiency is the set based on the IPPs because it shows larger delimitation of the infected regions and their borders.

Finally, we also studied a specimen of *V. vinifera* showing no pathologic symptomatology. As in the previous cases, we measured the experimental Mueller matrix and we retrieved the non-polarized transmission image (*M*_*00*_ in Fig. 7a) as a reference, and the polarimetric observables corresponding to the IPPs and CPs (Fig. 7b–g). In the non-polarized transmission image (Fig. 7a) we can observe two main leaf features, a vein (indicated by a blue arrow in Fig. 7a), and cell clusters (e.g., the green rectangle highlights a region of cell clusters in Fig. 7a). Importantly, polarimetric images in Fig. 8 allow the recognition of other plant structures not visible in non-polarimetric intensity images. This situation provides the importance of polarimetric channels for plant structures imaging, not only to increase visual image contrast, but also to reveal structures hidden in regular intensity images. In particular, contrarily to non-polarized intensity image (Fig. 7a), polarimetric images reveal the presence of a third structure consisting of a raphide. In vine leaves, raphides are made of calcium oxalate needle shaped crystals packed together^31,32^ forming prorated clusters of typically 80 µm (long axis) × 30 µm (short axes). Raphides are completely invisible in non-polarized transmission images (Fig. 7a), but their presence and spatial location becomes clearly visible in polarization-based images, for instance, they are well visible in the *P*_*1*_ image (Fig. 7b), indicated by pink arrows, and also, they are visible in the *P*, *D* and *P*_*S*_ images (Fig. 7e,f and g, respectively). In addition, the polarimetric analysis reveals another structure located at the upper-left part of the sample (indicated with white arrows in Fig. 7b and Fig. 7e and f, respectively) that has a polarimetric signature similar to that of the vein. This structure may correspond to a secondary vascular structure. From lower to higher, the mean values of the IPPs (Fig 7b–d) corresponding to the raphide are *P*_*1*_=0.24±0.01, *P*_*2*_=0.41±0.01 and *P*_*3*_=0.47±0.01, followed by these corresponding to the cell clusters: *P*_*1*_=0.47±0.01, *P*_*2*_=0.50±0.01 and *P*_*3*_=0.56±0.01, and finally the ones corresponding the leaf vein, *P*_*1*_=0.68±0.01, *P*_*2*_=0.72±0.01 and *P*_*3*_=0.81±0.01. Accordingly it can be said that the raphide possess an individual signature, different to that of the vein and the cluster of cells and therefore it can be said that there are three different classes in the image. The reasons that may explain the elevated depolarization of the raphide are the fluctuations in the polarimetric properties, and scattering, which may be higher in the raphide that in the vein or in the cluster of cells. Concerning scattering, it is expected that the refractive index mismatch between a given region and the surrounding media, at the origin of scatting, should be higher for the raphide, made of a solid inorganic component, than for veins or clusters of cells which are essentially made of a liquid similar to the surrounding media contained by the membranes forming the cell walls and other cell organelles. Importantly, note that *P*_*2*_ and *P*_*3*_ mean values in raphides and clusters of cells are quite similar to each other, but *P*_*1*_ is significantly different (0.24 and 0.47 for raphides and cell cluster, respectively), which explains why the raphides are clearly seen in the *P*_1_ image (Fig. 7b). The largest mean IPPs values correspond to the vein. This means that the vein structure induces little depolarization to the incident light, because it is a structure essentially filled with a liquid with low scattering and no polarimetric properties. Regarding the CPs observables, the highest polarizance (Fig. 7e) mean values are demonstrated for raphides (*P*=0.03±0.01), followed by the cell cluster (*P*=0.003±0.001) and the leaf vein (*P*=0.001±0.001). Likewise, the raphides show the highest mean diattenuation (*D*=0.07±0.01, Fig. 7f), to be compared to *D*=0.04±0.01 and *D*=0.02±0.01, corresponding to veins and clusters of cells respectively. Taking these values into account, the *V. vinifera* leaf shows low polarizance and diattenuation response in all its structures, and thus, it can be understood as a non-dichroic sample. Conversely, the highest mean values of the spherical purity *P*_*S*_ (Fig. 7g) are demonstrated for the leaf vein (*P*_*S*_=0.70±0.01), this being a direct consequence of the strong alignment of the cellulose filaments within the vein. In turn, the *P*_*S*_ values for the cell cluster are reduced to *P*_*S*_=0.49±0.01, and to *P*_*S*_=0.31±0.01 in the case of the raphides. In analogy to the image of the *P*_*1*_, these differences in the *P*_*S*_ mean values for the raphide, the cluster of cells and the vein, provide a well-contrasted image with well-differentiated regions (Fig. 7e).

The difference of the vein leaf sample with respect to the examples previously discussed is that it contains three classes instead of two of them. The latter may be a drawback to apply the pseudo-coloring based on the Euclidean distance, because it has been defined to handle only two classes. Therefore the pseudo-coloring based on Normal distribution may have an advantage in this particular situation. Accordingly and unlike in the examples previously discussed, we select three classes of structures to be simultaneously visualized (in the Normal-based approach): the raphides, a cell cluster and the leaf vein. The selected regions of interest, corresponding to the three classes, are indicated with pink, lime-green and blue squares within the purity index *P*_*1*_ (Fig. 8a). In the case of the Euclidean distance method, as it is restricted to handle only two classes, the selected classes are the raphide (in pink) and the cell cluster (lime-green). Note that unlike in the previous samples, instead of using the non-polarized transmission image (*M*_*00*_; Fig. 7a) to design the ROIs for the classes we use the *P*_*1*_ image (Fig. 8a) since the raphides are not visible in the non-polarized transmission intensity channel. Implemented pseudo-colored images are provided in Figs. 8b-8e, for Euclidian distance (IPPs based in Fig. 8b and CPs based in Fig. 8d) and for the Normal-based models (IPPs based in Fig. 8c and CPs based Fig. 8e). Concerning the performance of the two methods in terms of class coloring and visual discrimination, we observe some differences. On the one hand, the IPPs are not sensitive enough to correctly identify the location of raphides when implementing the Euclidean approach (Fig. 8b). In particular, some pixels belonging to raphides are not well-colored in pink, but other pixels that do not belong to raphides, they are incorrectly painted in pink. Unlike this, the Euclidean method applied with CPs observables is much more efficient and quite accurate discriminating between raphides and cell cluster (Fig. 8d). On the other hand, the IPPs observables applied with the Normal-based approach are able to correctly identify and localize all the studied classes: raphides (pink regions in Fig. 8c), the leaf vein (blue region in Fig. 8c) and the cell cluster (lime-green pixels in Fig. 8c). Furthermore, other structure previously discussed in polarization images (Fig. 7), the vascular structure located on the upper-left part of the sample image, it is also colored in blue (indicated with a white arrow in Fig. 8c), as it is recognized as part of a vein (as previously told, vascular structure and vein presents very similar polarimetric response, and therefore they are recognized as part of the same class). Finally, when applying the CPs observables with the Normal-based approach (Fig. 8e), all the classes are correctly discriminated as well, but due to they are more affected by distances between pixel-values and mean classes-values, when applying the Gaussian probability function (Eq. (9) in “Methods” section), more pixels tends to zero probability of belonging to any class, and then, painted in black, this darkening the whole image.

Summarizing, due to the excellent recognition and visualization of the classes, as well as the capability of discriminating more than two classes simultaneously (three in this case), the best results for the *V. vinifera* sample in terms of visual structure discrimination are obtained when applying the Normal-based approach, implemented with the IPPs observables (Fig. 8c). Importantly, if one is just interested to discriminate between two classes, the Euclidean distance method based on CPs also provides excellent results (Fig. 8d).

As a final remark, we want to highlight that the present work provides the suitability of implementing two robust polarimetric image-processing methods, the Euclidean distances and the Normal (Gaussian) function (described in “Methods” section), for the visual enhancement of image contrast and the higher accuracy in spatial location of the different biological structures within the inspected samples. Both methods are based on the association of different colors with the particular tissue classes that should be highlighted, or discriminated, within the sample, leading to pseudo-colored images including information of a group of different polarimetric observables into a single image. The two presented approaches can be used to develop automatic coloring methods, and surpass proposals previously presented in the literature, which were based on very basic linear combinations of polarimetric observables whose weights were obtained with a non-optimal and mostly heuristic approach^1,24,25^. Going more specifically to the obtained results, when applying these two methods to discriminate among different structures present in organic samples (lamb trachea, lamb tongue, a *Q. pubescens* leaf and a *V. vinifera* leaf) they have revealed different strengths and drawbacks. The Euclidean method provided very good results to discriminate between two different classes in the studied samples (see for instance, Figs. 2b, 4d, 6b and 8d) but as it has been said, this method is restricted to the study of only two classes. In turn, the major strengths of the Normal-based approach are that it can provide discrimination between more than two classes (see for instance Fig. 8c) and that it provides a further interpretation, as it is based on the probability of a given pixel to belong to a given class. The Normal based approach can also be used as an automatic classifier. However, the pseudo-coloring Normal approach is very sensitive to the actual statistical distribution, the variance and the presence of outliers in the data. The Normal distribution approach works well when data is normally or close to normally distributed without outliers. In real-life data, departures from the ideal normal distribution in data values results in either misclassification or black pixels, i.e. failure of identification to a given class (see for instance Fig. 2c and e).

In this work, we have provided optimized pseudo-colored functions based on polarimetric spaces, which enhance other approaches we previously presented in literature^1,24,25,30^. However, we want to note that the application of pseudo-colored functions is not limited to polarimetric spaces^33–36^, and there exist other approaches, as image segmentation and coloring approaches^37–40^, that lead to interesting results in terms of structures visualization. These approaches can be applied directly on polarimetric images, the resulting methods favoring from the inherent image enhancement of polarimetry images, but further comparison must be conducted in order to determine the best possible pseudo-colored imaging scenario. Regarding to the application of the IPPs or the CPs observables within the proposed methods, we have chosen these metrics because previous works highlight the suitability of depolarization observables for the discrimination of tissues^1,4,5,11,16,28^, and because these two bases, all together, completely describe the depolarization response of a samples^27^. Therefore, IPPs and CPs represent an ideal framework to implement pseudo-colored functions for tissue discrimination. Regarding the samples discussed in this section, we have demonstrated that depending on the characteristics of the particular sample, either IPP or CP basis, provide to excellent visualization of the structures when used to apply pseudo-coloring strategies. Accordingly, for each sample to be analyzed, we recommend the use to the two bases of observables, i.e. the IPPs and CPs. Whereas IPPs are sensitive to the structure of the depolarization in samples (i.e., depolarization anisotropies), the CPs are more related to the physical properties of the constituents of the sample being at the roots of depolarization (retardance, polarizance, diattenuation).

At the end, the characteristics of each studied sample will determine which one of the two basis will provide the more vivid contrast.

## Methods

### Sample description

The animal samples used in this study were a section of a trachea and a section of a tongue dissected from an ex-vivo lamb sample bought from a grocery store. All experimental protocols were carried out in accordance with relevant guidelines and regulations. The two vegetal samples used in this work were (1) a leaf of *Q. pubescens* specimen infected with *E. alphitoides*, which causes powdery mildew lesions on leaf surface and (2) a leaf of *V. vinifera* specimen showing no symptoms of disease. The *Q. pubescens*, a species of white oak, belongs to the Fagaceae family and it is commonly found in central and southern Europe. It produces acorns (oak nuts) which can be consumed or extract their oil. *V. vinifera*, commonly known as grape vine, belongs to the Vitaceae family. Native from the central Europe, the land regions around the Mediterranean Sea and southwestern Asia, *V. vinifera* is cultivated worldwide for both grape (fresh or dried) consuming, and vinegar and wine production.

The *Quercus pubescens* leaf used in this study was kindly provided by Dra. Teresa Garnatje (Botanical Institute of Barcelona (IBB, CSIC-ICUB), Barcelona, Spain) and Dr. Jordi Luque (Institute of Agrifood Research and Technology (IRTA), Cabrils, 08348, Spain). The *Vitis vinifera* leaf was kindly provided by Dr. E. Garcia-Caurel. All the sample collecting methods were performed in accordance with relevant guidelines and regulation. T. Garnatje and J. Luque undertook the formal identification of the plant material used in this study. An herbarium voucher of *Q. pubescens* is deposited in the Herbarium of the Botanical Institute of Barcelona (BC-983018).

### Polarization observables

Among the wide variety of mathematical approaches^21^, the Mueller-Stokes (M-S) formalism is especially suitable for the description of the polarimetric properties of turbid media, as it is based on radiometric measurements and allows to deal with the depolarization content of samples^22,23^. In this approach, the state of polarization of light beams is characterized by the so-called Stokes vector (S) and the polarimetric characteristics of the sample are complexly encoded into the 4 × 4 real matrix called Mueller matrix (MM). The generic MM block form is defined as:

1 $$M = m_{00} \left( {\begin{array}{*{20}c} 1 & {D^{T} } \\ P & m \\ \end{array} } \right).$$

From the structure of the MM (Eq. (1)) we can easily retrieve the non-polarized transmission or reflection (*m*_*00*_) and the dichroism (diattenuation and polarizance, the 3-dimensional vectors *D* and *P*, respectively). However, the polarimetric properties related to the retardance and the depolarization are entangled in a 3 × 3 submatrix, *m.* Concerning to the polarimetric observables, whereas the diattenuation gives a measure of the transmission /reflection dependence of the sample with the input polarization state, polarizance describes the capability of the sample to polarize a fully unpolarized input light beam. In addition, the degree of spherical purity, *P*_*S*_, defines the portion of depolarization which is not directly related with dichroic properties of the sample.

2 $$D = \frac{{\sqrt {m_{01}^{2} + m_{02}^{2} + m_{03}^{2} } }}{{m_{00} }},\quad P = \frac{{\sqrt {m_{10}^{2} + m_{20}^{2} + m_{30}^{2} } }}{{m_{00} }},\quad P_{S} = \frac{{\left\| m \right\|_{2} }}{\sqrt 3 },$$

where $\left\| m \right\|_{2}$ is the 2-norm of the sub-matrix *m*. To retrieve the depolarization content, we conduct the Cloude’s decomposition^21^ which defines the MM as a parallel combination (i.e., convex sum) of four non-depolarizing (pure) MMs, labeled as *M*_*Ji*_, whose statistical weights are proportional to the covariance matrix *H(M)* eigenvalues (*λ*_*i,*_)^22^:

3 $$M = m_{00} \sum\limits_{i = 0}^{3} {\hat{\lambda }_{i} } \hat{M}_{Ji} ,\quad \lambda_{0} \ge \lambda_{1} \ge \lambda_{2} \ge \lambda_{3} \ge 0$$

Interestingly, the depolarizing response being synthetized within the four pure components *M*_*Ji*_ allows to retrieve the different types of depolarizers by simply looking at the weights corresponding to each pure component. The combination of the above-mentioned normalized eigenvalues results into the definition of the Indices of Polarimetric Purity (IPPs)^26^: three real, dimensionless and invariant parameters which provide information about the polarimetric randomness induced by the sample to input polarization states. The IPPs are defined as:

4 $$P_{1} \equiv \hat{\lambda }_{0} - \hat{\lambda }_{1} ,\quad P_{2} \equiv \hat{\lambda }_{0} + \hat{\lambda }_{1} - 2\hat{\lambda }_{2} ,\quad P_{3} \equiv \hat{\lambda }_{0} + \hat{\lambda }_{1} + \hat{\lambda }_{2} - 3\hat{\lambda }_{3} ,$$

and are restricted to *0* ≤ *P*_*1*_ ≤ *P*_*2*_ ≤ *P*_*3*_ ≤ *1*. The IPPs define a real 3D-depolarization space whose interpretation is related to the polarimetric randomness (i.e., depolarization) induced by different mechanisms within the sample. Accordingly, the IPPs’ depolarization space constitutes a suitable tool to discriminate among structures with different depolarization signatures due to their inherent components. Recalling that the depolarizing response of a sample can be synthetized as the incoherent sum of four pure components (*M*_*Ji*_ in Eq. (3)), the IPPs correspond to the statistical weight corresponding to each of these pure components^22,26^. In particular, *P*_*1*_ is associated with the portion of the pure non-depolarizing component, *P*_*2*_*-P*_*1*_ quantifies the statistical portion of a bidimensional depolarizer, *P*_*3*_*-P*_*2*_ corresponds to the portion of a tridimensional depolarizer (equiprobable mixture of three pure components) and *1-P*_*3*_ quantifies the statistical weight of an ideal depolarizer. Furthermore, the depolarization index, *P*_*Δ*_, estimates the overall depolarization of the MM. Importantly, this observable allows to connect the polarimetric spaces of (1) the Components of Purity (CPs; composed by the enpolarization metrics corresponding to the diattenuation *D*, polarizance *P* and the degree of spherical purity *P*_*S*_) and (2) the Indices of Polarimetric Purity (IPPs; *P*_*1*_*, P*_*2*_ and *P*_*3*_) in the following way:

5 $$P_{\Delta } = \frac{1}{\sqrt 3 }\sqrt {D^{2} + P^{2} + 3P{}_{S}^{2} } = \frac{1}{\sqrt 3 }\sqrt {2P_{1}^{2} + \frac{2}{3}P_{2}^{2} + \frac{1}{3}P{}_{3}^{2} } ,\quad \quad 0 \le P_{\Delta } \le 1$$

Importantly, when *P*_*1*_ = *P*_*2 *_= *P*_*3 *_= *P*_*Δ *_= *1* is found, the indicators characterize a non-depolarizing (pure) system. Conversely, the ideal depolarizer is defined by *P*_*1 *_= *P*_*2 *_= *P*_*3 *_= *P*_*Δ *_= *0.*

### Pseudo-coloring approaches

In the following, we define the parameters of interest involved in the pseudo-coloring models, both for the Euclidean and Normal cases. Assume, from the experimental Mueller matrix (MM) measurement of a given sample, the extraction of *n* MM-derived polarimetric observables, $\overrightarrow {p} = [p_{1} ,...,p_{n} ]$^21^. Note that the polarimetric observables, $\overrightarrow {p}$, we use in this study are those defined in the previous section, i.e., the Components of Purity, CPs, and the Indices of Polarimetric Purity, IPPs. Additionally, consider the definition of *i* classes corresponding to different organic tissues. Each kind (*i* = 1, …, *k*) of organic tissues (trachea ring, trachea sheath, tongue papillae tissue, tongue epithelial tissue, *Q. pubescens* powdery mildew, *Q. pubescens* lamina, *V. vinifera* vein, *V. vinifera* raphides and *V. vinifera* cell cluster; in our study) is characterized by the *j* = 1, …, *n* means,$m_{j}^{i}$, and standard deviations, $\sigma_{j}^{i}$, corresponding to the $p_{j}^{i}$ observables calculated from a Region of Interest (ROIs) within the specific class *i*. In addition, for image coloring purposes, we also define the vector $\vec{C}^{i} = \left[ {R^{i} ,G^{i} ,B^{i} } \right]$ with *i* = 1, …, *k* as the standard RGB color space triplet associated with a particular class *i*.

### Euclidean distances for *k=2* classes

In this case we assume we only need to discriminate between *k* = *2* classes (e.g. healthy/infected tissue). This pseudo-colored approach is based on the Euclidean distance from the values of $\overrightarrow {p}$ from a given image pixel to the mean values $\overrightarrow {{m^{i} }} = [m_{1}^{i} ,...,m_{n}^{i} ]$ of the n polarimetric observables of a given class (*i* = *1* or *i* = *2*). The normalized distance $d^{i,norm}$ is given by,

6 $$d^{i,norm} = \sqrt {\sum\limits_{j = 1}^{n} {\left( {\frac{{d^{i} }}{{m_{j}^{i = 1} - m_{j}^{i = 2} }}} \right)^{2} } } = \sqrt {\sum\limits_{j = 1}^{n} {\left( {\frac{{m_{j}^{i} - p_{j} }}{{m_{j}^{i = 1} - m_{j}^{i = 2} }}} \right)^{2} } } ,\quad i = 1,2,$$

where $m_{j}^{i = 1} - m_{j}^{i = 2}$ corresponds to the distance between the means of the two classes for a given polarimetric observable, *p*_*j*_ (*j* = *1, …, n*). Importantly, we associate a given color, $\overrightarrow {{C_{i} }}$, to each particular class, i. Consequently, the larger the distance from a given pixel to a given class, the lower the corresponding weight to its particular class color, $\overrightarrow {{C_{i} }}$. Accordingly, each particular class color $\overrightarrow {{C_{i} }}$ is pondered by the following subtraction,

7 $$\Re^{i} = 1 - \frac{{d^{i,norm} }}{d},\quad i = 1,2,$$

where $d^{i,norm}$ is normalized by the sum of the distances (i.e., $d = d^{i = 1,norm} + d^{i = 2,norm}$), so that the subtraction is positive-definite and ranges between 0 and 1. Thus, the larger the distance, $d^{i,norm}$, the lower the amount of i-color level included in the pixel. The final pixel color for the two classes (*i* = 1, 2), is given by,

8 $$\vec{C} = \left[ {R,G,B} \right] = \Re^{1} \left[ {R^{1} ,G^{1} ,B^{1} } \right] + \Re^{2} \left[ {R^{2} ,G^{2} ,B^{2} } \right].$$

Importantly, the method does not behave as a classifier. Note that some pixels within the image may contain more than one color, thus showing a mixed tone between the two basis (pure) colors. Moreover, there are some pixels that correspond to some parts of the sample which do not belong to any inspected class, either *i* = *1* nor *i* = *2*. In such case, we can equally interpret both distances (i.e., $$d^{i = 1,norm} = d^{i = 2,norm}$$), and thus, the $${\raise0.7ex\hbox{${d^{i,norm} }$} \!\mathord{\left/ {\vphantom {{d^{i,norm} } d}}\right.\kern-\nulldelimiterspace} \!\lower0.7ex\hbox{$d$}}$$ term in Eq. (7) tends to 1/2 so that *ℜ*^*i *^*≈ 1/2*. Under this approach, as both *ℜ*^*i*^ tend to be 1/2, the resulting color for these pixels is an equal mixture of the two selected colors. Therefore, the resulting color encoding cannot be used for classificatory proposes, but just for visual enhancement of the images as shown in “Results” and “Discussion” sections. Pseudo-coloring based on the Euclidean-distances method is very efficient in terms of tissues visualization and discrimination.

### Normal distribution for *k* classes

The previous method is difficult to generalize to more than two classes (*k*>2). For this reason, in this subsection we propose a second method to construct pseudo-colored functions, in this case, based on the Normal (Gaussian) probability distribution of the *n* polarimetric observables. Contrary to the above-presented Euclidean method, the Normal approach allows to inspect an unlimited number of features (i.e., classes), *k*. The probability *P*^*i*^ of a pixel corresponding to the *jth* observable (*j*=*1,..,n*) of being part of a given class *i* (*i*=*1,..,k*) is defined as,

9 $$P^{i} = \prod\limits_{j} {\exp \left[ { - \left( {\frac{{p_{j} - m_{i,j} }}{{\sigma_{i,j} }}} \right)^{2} } \right]} ,$$

which is limited to range between 0 and 1. Therefore, for each pixel we get as many probability functions, *P*^*i*^, as classes, *i*, we deal with*.* Afterwards, the pseudo-colored image is constructed by associating each standard RGB color space triplet to their corresponding probability function (*P*^*i*^) in the following way:

10 $$\vec{C} = \left[ {R,G,B} \right] = \sum\limits_{i} {P^{i} \cdot C}^{i} = \sum\limits_{i} {P^{i} \left[ {R^{i} ,G^{i} ,B^{i} } \right]} .$$

This approach outputs a colored polarimetric image, $\overrightarrow {C} = [R,G,B]$, which is based on the linear combination of the *P*^*i*^* ·C*^*i*^ terms of the *i* classes involved. In other words, the amount of *i*-color level within a pixel is pondered by the probability of the particular pixel to be recognized as belonging to the class *i*. Therefore, unlike the Euclidean distance approach, the pixel color coding associated to this Normal-based model can be used for classificatory proposes.

### Complete image Mueller polarimeter

The experimental Mueller matrices images of tissues are acquired by means of a complete image Mueller polarimeter consisting of two independent and mobile arms, the Polarization State Generator (PSG) and the Polarization State Analyzer (PSA), which are capable to generate and analyze, respectively, any fully polarized state. The optical systems comprising both PSG and PSA are based on Parallel Aligned Liquid Crystals (PA-LC, Variable Retarders with Temperature Control) retarders (LVR-200-400-700-1LTSC distributed by Meadowlark Optics). In particular, the PSG is composed by a linear polarizer (Glan-Thompson prism-based CASIX) oriented at 0°, followed by two PA-LC oriented at 45° and 0°. Likewise, the PSA optical set-up is composed by the same optical elements as the PSG but arranged in reverse order. The polarizer within the PSA corresponds to a dichroic sheet polarizer distributed by Meadowlark Optics. All orientations are with respect to the laboratory vertical. The light source device is a four-wavelength high-power Thorlabs LED source (LED4D211, operated by DC4104 drivers distributed by Thorlabs). It is placed on the PSG and allows to illuminate with different wavelengths covering the visible spectrum (from 400 to 700 nm, approx.). Complementary, we use 10 nm dielectric bandwidth filters distributed by Thorlabs: FB530-10 and FB470-10 for green and blue wavelengths, respectively. The image acquisition (i.e., sample intensity) is conducted by means of the set comprising a 35 mm focal length Edmund Optics TECHSPEC® high resolution objective followed by an Allied Vision Manta G-504B CCD camera, with 5 Megapixel GigE Vision and Sony ICX655 CCD sensor (of 2452(H) × 2056(V) resolution and cell size of 3.45 × 3.45 μm). Both optical components are placed on the PSA system, achieving a spatial resolution of 22 μm.

The Mueller matrix measurements of the lamb trachea, the lamb tongue and the *Q. pubescens* are conducted at 470 nm illumination wavelength by tilting by 34° the PSG with respect to the laboratory horizontal reference and holding the PSA at 0°. This configuration allows us to avoid the ballistic reflection. From the whole sample, we select a region of interest (ROI) of 512 × 512 pixels, thus corresponding to an area of 1.1 × 1.1 cm^2^. With regards to the experimental Mueller matrix of the *V. vinifera* leaf, it is acquired by means of a multimodal microscope polarimeter^3^ coupled to a light source of white light LED with a narrow-band spectral filter of 533 nm and a spectral width of 15 nm. The microscope is set at transmission configuration, where the sample leaf is placed between two identical microscope objectives (for imaging and illumination, respectively). The achieved magnifications are of 50X, 20X, or 5X.

## Supplementary Information

Supplementary Information.

## Data Availability

The datasets generated during and/or analysed during the current study are not publicly available due to the conduction of different research studies but are available from the corresponding author on reasonable request.
