# Supplementary material for: Automatic pseudo-coloring approaches to improve visual perception and contrast in polarimetric images of biological tissues
Source: Sci Rep. 2022 Nov 2;12:18479. doi: 10.1038/s41598-022-23330-6 (PMC9630374; doi:10.1038/s41598-022-23330-6)
Supplement: Supplementary file 1 — Supplementary Information. [file 41598_2022_23330_MOESM1_ESM.docx]

Automatic pseudo-coloring approaches to improve visual perception and contrast in polarimetric images of biological tissues

**Carla Rodríguez**1,***, Albert Van Eeckhout**1,2**, Enrique Garcia-Caurel**2**, Angel Lizana**1 **and Juan Campos**1

1Optics group, Physics Department, Universitat Autònoma de Barcelona, Bellaterra, 08193, Spain

2LPICM, CNRS, Ecole Polytechnique, Institut Polytechnique de Paris, Palaiseau, 91120, France

*[carla.rodriguez@uab.cat](mailto:carla.rodriguez@uab.cat)

1. **Polarimetric observables for biological samples inspection**

This section is devoted to show the suitability of using polarimetric observables for biological tissue inspection. In the following we show the polarimetric images corresponding to the non-polarized intensity (M_00_), the indices of polarimetric purity (IPPs: P_1_, P_2_ and P_3_), the depolarization index P_Δ_, the components of purity (CPs: P, D and Ps) and the global retardance R, from a set of four different biological samples. In particular, we inspect the lamb trachea section and the *Vitis vinifera* plant sample, already shown in the manuscript. In addition, we provide the polarimetric images taken from a leaf of *Prunus dulcis* plant specimen showing disease symptoms from fungal infection and a section of a lamb heart.

Figure S1 shows the polarimetric images from the lamb trachea section. The cartilaginous rings and the trachea sheath is clearly distinguished by the enpolarization metrics of *P_2_*, *P_3_*, *P*, and *P_S_* (Figs. S1c, S1d, S1f and S1h), as they point well the difference between the two types of tissues and also present an enhanced contrast when compared to the non-polarized diffuse reflectance (*M_00_* in Fig. S1a).


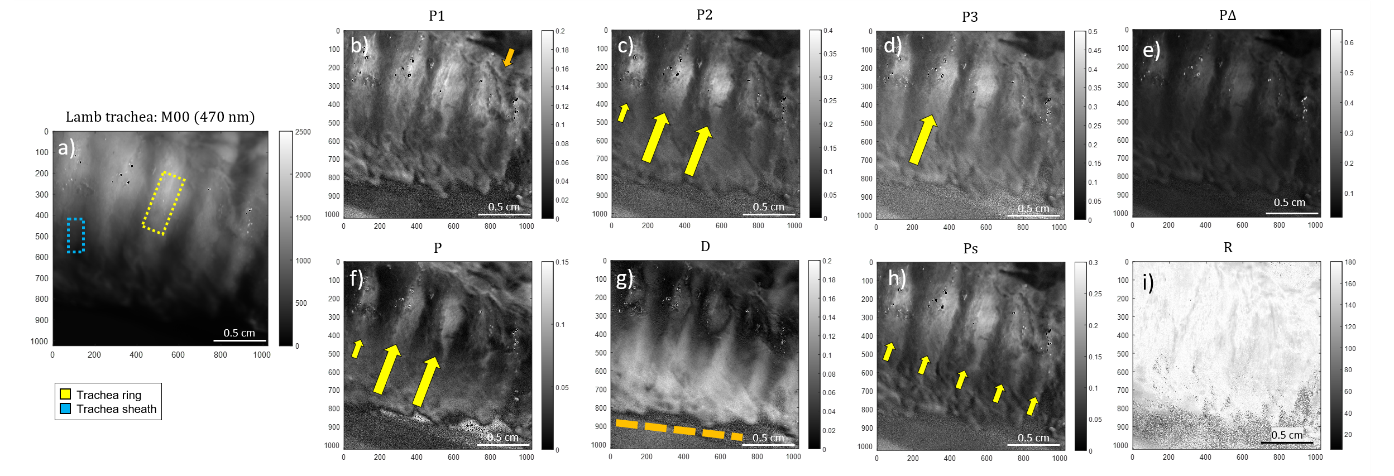


**Figure S1**. Polarimetric images of a lamb trachea measured at 470 nm illumination wavelength: a) unpolarized intensity image (*M_00_*), the Indices of Polarimetric Purity b) *P_1_*, c) *P_2_* and d) *P_3_*, the depolarization index e) P_Δ_, the Components of Purity f) *P*, g) *D* and h) *P_S_* and i) the global retardance R. The yellow dotted box (Fig. S1a) and yellow arrows (Figs. S1c, S1d, S1f and S1h), indicate the location of the cartilaginous rings. The dotted blue box (Fig. S1a) indicates the location of the trachea sheath. The orange arrow (Fig. S1b) and the orange dotted line (Fig. S1g) show the vascular structure within the external trachea sheath and the sample border, respectively.

To highlight the behavior of the polarimetric observables, we computed the visibility, $V=\left[I_{\max}-I_{\min}\right]/\left[I_{\max}+I_{\min}\right]$ (see Table S1) of the sample regions corresponding to the trachea rings and the trachea sheath for each of the metrics here discussed. Additionally, we present the subtraction of the visibility value between both structures. The largest visibility difference between the lamb trachea rings and sheath is demonstrated by the diattenuation D (0.4753 in Table S1), followed by the index of polarimetric purtiy P_3_ and the depolarization index P_Δ_ (0.2621 and 0.2363 in Table S1, respectively).

**Table S1.** Visibility of different polarimetric observables corresponding to the trachea ring and trachea sheath in lamb trachea sample. The largest differences in visibility values between the inspected structures is highlighted in gray.

|  | | ***M_00_*** | ***P_Δ_*** | ***P_1_*** | ***P_2_*** | ***P_3_*** | ***P*** | ***R*** | ***D*** | ***P_S_*** |
| --- | --- | --- | --- | --- | --- | --- | --- | --- | --- | --- |
| *Lamb trachea* | Ring | 0.2596 | 0.4639 | 0.4258 | 0.5644 | 0.5130 | 0.6974 | 0.1030 | 0.9434 | 0.4542 |
|  | Sheath | 0.3145 | 0.2276 | 0.4599 | 0.3859 | 0.2509 | 0.5782 | 0.1348 | 0.4681 | 0.4168 |
| Subtraction:  Ring *vs* Sheath | | 0.0549 | 0.2363 | 0.0341 | 0.1785 | 0.2621 | 0.1192 | 0.0318 | 0.4753 | 0.0374 |

Similar analysis is conducted for the *V. vinifera* plant sample. Figure S2 shows the polarimetric images from *V. vinifera* specimen. Importantly, raphides are completely invisible in non-polarized transmission images (i.e., M_00_ in Fig. S2a), but their presence and spatial location becomes clearly visible in polarization-based images, for instance, they are clearly visible in the index of polarimetric purtiy *P_1_* image (Fig. S2b), indicated by pink arrows, and also, they are visible in the *P_Δ_*, *P*, *D* and *P_S_* images (Figs. S2e-S2h, respectively).


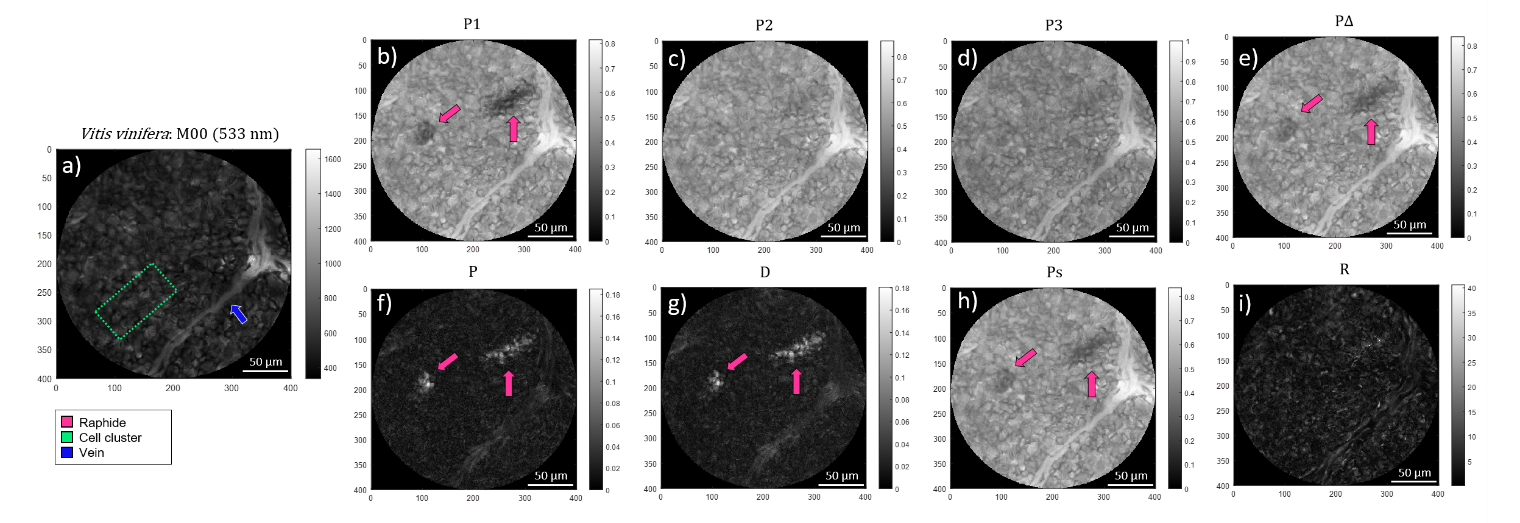


**Figure S2.** Polarimetric images of the *Vitis vinifera* leaf measured under the microscope for 533 nm illumination wavelength: a) non-polarized transmission intensity image (*M_00_*), the Indices of Polarimetric Purity b) *P_1_*, c) *P_2_* and d) *P_3_*, the depolarization index e) P_Δ_, the Components of Purity f) *P*, g) *D* and h) *P_S_* and i) the global retardance R. The blue arrow (Figs. S2a) indicates the location of the vein. The pink arrows (Figs. S2b, S2e-S2h) indicate the location of the raphides. The lime-green dotted box indicates an illustrative region comprising a cell cluster.

Likewise, the visibility values corresponding to the raphides and the cell cluster are presented in Table S2 as well as the subtraction between their values. The largest visibility difference between the raphide and the cell cluster is achieved by the index of polarimetric purity *P_1_* (the 0.3154 In Table S2), followed by polarizance, *P,* and diattenuation, *D* (0.1269 and 0.1234 in Table S2, respectively).

**Table S2.** Visibility of different polarimetric observables corresponding to the raphide and the cell cluster in *Vitis vinifera* plant sample. The largest difference in visibility values between the inspected structures is highlighted in gray.

|  | | **M_00_** | **P_Δ_** | **P_1_** | **P_2_** | **P_3_** | **P** | **R** | **D** | **P_S_** |
| --- | --- | --- | --- | --- | --- | --- | --- | --- | --- | --- |
| *V. vinifera* | Raphide | 0.2319 | 0.2829 | 0.5410 | 0.2323 | 0.2305 | 0.9059 | 0.9698 | 0.8727 | 0.2963 |
|  | Cell cluster | 0.2312 | 0.2192 | 0.2256 | 0.2029 | 0.2484 | 0.7790 | 0.9763 | 0.7493 | 0.2195 |
| Subraction:  Raphide vs Cell cluster | | 0.0007 | 0.0637 | 0.3154 | 0.0294 | 0.0179 | 0.1269 | 0.0065 | 0.1234 | 0.0768 |

Additionally, in Fig. S3 we show the polarimetric images corresponding to a 2.2 x 2.2 cm^2^ section of a lamb heart. In particular, the different polarimetric properties of the sample allow the enpolarizing metrics to reveal different structures. That is the case of small vascular structures within the lamb heart revealed by the Index of Polarimetric Purity, P_1_, and the polarizance, P (yellow arrows in Figs. S3b and S3f, respectively). Moreover, the roughness pattern of the tissue is enhanced by Index of Polarimetric Purity, P_2_ and the degree of spherical purity P_S_ (green arrows in Figs. S3c and S3h, respectively). Retardance R (Fig. S3i) also reveals some vascular structures and the striated tissue region (yellow and green arrow in Fig. S3i, respectively). However, this polarimetric observable does not enhance the overall image contrast as much as the depolarizing metrics of the Indices of Polarimetric Purity (P_1_, P_2_ and P_3_) and the degree of spherical purity P_S_ do (Figs. S3b-S3d and S3h, respectively).


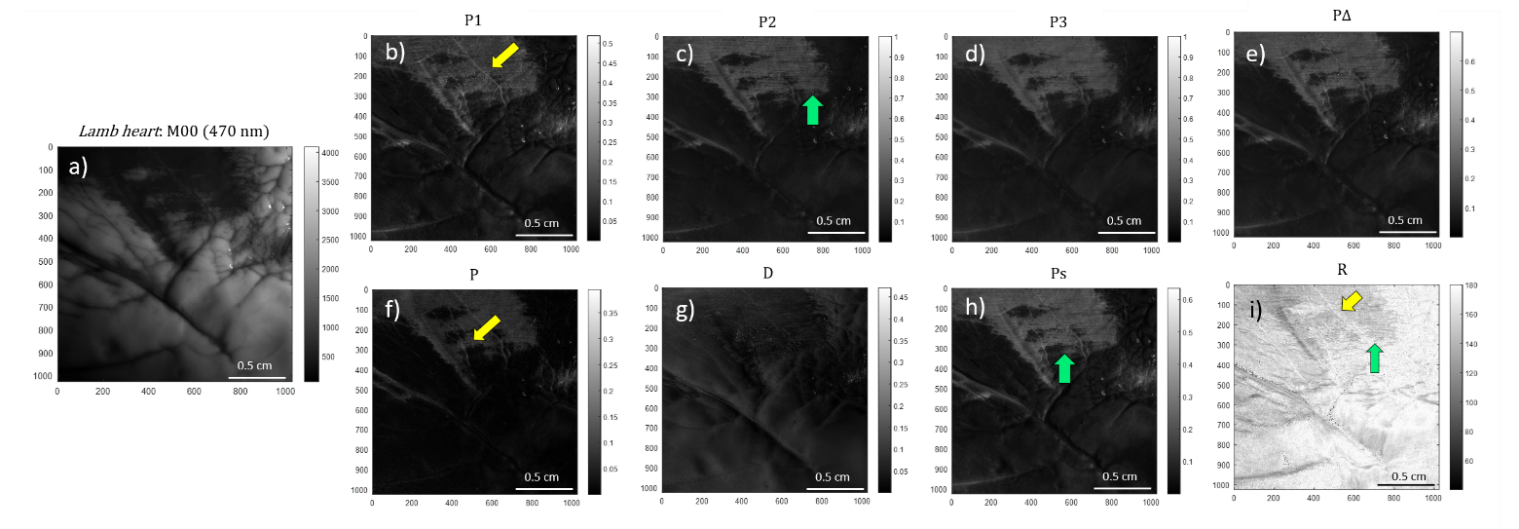


**Figure S3.** Polarimetric images of the lamb heart measured at 470 nm illumination wavelength: a) non-polarized transmission intensity image (*M_00_*), the Indices of Polarimetric Purity b) *P_1_*, c) *P_2_* and d) *P_3_*, the depolarization index e) P_Δ_, the Components of Purity f) *P*, g) *D* and h) *P_S_* and i) the global retardance R. The yellow arrows (Fig. S3b, S3f and S3i) indicate the location of a particular vascular structure. The green arrows (Fig. S3c, S3h and S3i) indicate the region showing different roughness.

Finally, in Fig. S4 we show the polarimetric images corresponding to a leaf of *Prunus dulcis* plant specimen showing disease symptoms from fungal infection. The disease is mostly located within the dark spot located at the down-left part of the intensity image M_00_ (indicated with the pink arrow in Fig. S4a). The depolarization-related observables (i.e., P1, P2 and P3 and P_Δ_ in Figs. S4b-S4e, respectively) as well as the Components of Purity (i.e., P, D and Ps in Figs. S4f-S4h) reveal the small vascular structures contained inside the spot (pink arrow in Fig. S4b), which are unable to be detected by means of regular intensity image (Fig. S4a). On the contrary, retardance R focuses on the principal vein (Fig. S4i).


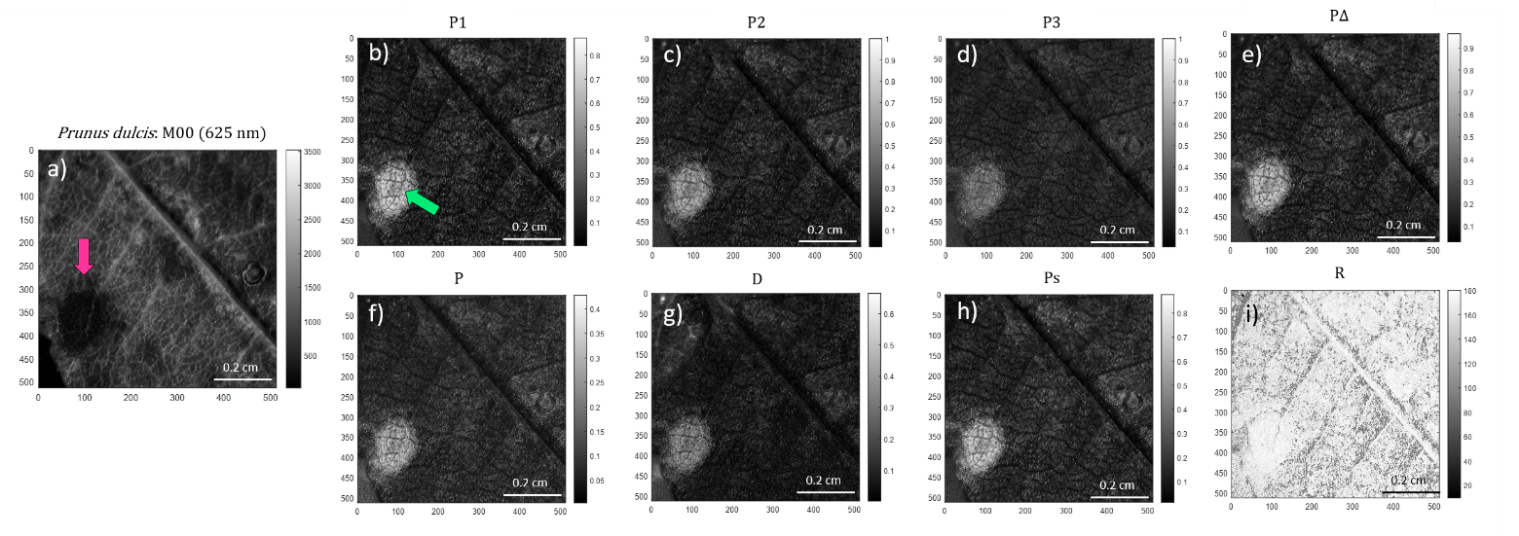


**Figure S4.** Polarimetric images of the *Prunus dulcis* leaf measured at 625 nm illumination wavelength: a) non-polarized transmission intensity image (*M_00_*), the Indices of Polarimetric Purity b) *P_1_*, c) *P_2_* and d) *P_3_*, the depolarization index e) P_Δ_, the Components of Purity f) *P*, g) *D* and h) *P_S_* and i) the global retardance R. The pink and green arrows (Figs. S4a and S4b, respectively) indicate the location of the fungal disease and the small vascular structures contained.
